# Simultaneous gene editing of both nuclei in a dikaryotic strain of *Ganoderma lucidum* using Cas9-gRNA ribonucleoprotein

**DOI:** 10.71150/jm.2409006

**Published:** 2025-01-24

**Authors:** Yeon-Jae Choi, Hyerang Eom, Rutuja Nandre, Minseek Kim, Youn-Lee Oh, Sinil Kim, Hyeon-Su Ro

**Affiliations:** 1 Department of BioMedical Bigdata (BK21) and Research Institute of Life Sciences, Gyeongsang National University, Jinju 52828, Republic of Korea; 2 Mushroom Science Division, National Institute of Horticultural and Herbal Science, Rural Development Administration, Eumseong 27709, Republic of Korea; 3 Biological Resources Utilization Division, National Institute of Biological Resources (NIBR), Incheon 22689, Republic of Korea

**Keywords:** *Ganoderma*, mushroom, gene editing, dikaryotic, heterokaryosis

## Abstract

The presence of multiple nuclei in a common cytoplasm poses a significant challenge to genetic modification in mushrooms. Here, we demonstrate successful gene editing in both nuclei of a dikaryotic strain of *Ganoderma lucidum* using the Cas9-gRNA ribonucleoprotein complex (RNP). The RNP targeting the *pyrG* gene was introduced into dikaryotic protoplasts of *G. lucidum*, resulting in the isolation of 31 mycelial colonies resistant to 5-fluoroorotic acid (5-FOA). Twenty-six of these isolates were confirmed as dikaryotic strains by the presence of two distinct *A* mating type markers, denoted as *A1* and *A2*. All dikaryons exhibited clamp connections on their mycelial hyphae, while the remaining 5 transformants were monokaryotic. Subsequent sequence analysis of PCR amplicons targeting *pyrG* revealed that two dikaryons harbored disrupted *pyrG* in both nuclei (*pyrG*-/*pyrG*-), while 10 and 14 displayed *pyrG*+/*pyrG*- (A1/A2) and *pyrG*-/*pyrG*+ (A1/A2) configurations, respectively. The disruption was achieved through non-homologous end joining repair, involving deletion or insertion of DNA fragments at the site of the double-strand break induced by RNP. Importantly, the nuclei were stable throughout 10 serial transfers over a period of 6 months. These findings highlight the capability of RNP to target genes across multiple nuclei within the same cytoplasm.

## Introduction

Filamentous fungi typically exhibit multinucleate cells where multiple nuclei share a common cytoplasm. This characteristic is also observed in mushrooms, which are dikaryotic septate fungi primarily belonging to Basidiomycota. Contrary to conventional belief, dikaryotic fungi do not always contain only two nuclei per cytoplasm; rather, they harbor multiple nuclei of two distinct types. For instance, it has been reported that dikaryotic cells in the fruiting body of *Agaricus bisporus* can possess anywhere from 5 to 9 nuclei per cell (Craig et al., 1979), while mycelial cells may contain up to 25 nuclei within a single cytoplasm (Gehrmann et al., 2018; Saksena et al., 1976). This phenomenon of heterokaryosis is also observed in monokaryotic strains of *A. bisporus*, as demonstrated in our previous study (Choi et al., 2023a). Accordingly, the first step in genetic studies of these higher fungi entails obtaining a monokaryotic strain with a nucleus of singular genetic origin, accomplished through a de-dikaryotization process involving protoplast generation, spreading on solid medium, isolation of mycelial colonies, and examination of nuclei within the mycelial cells (Ha et al., 2019; Miyazaki et al., 2000).

Besides the complexity of heterokaryosis, the lack of suitable molecular biological tools has impeded genetic modifications in mushrooms. The recent integration of gene editing technologies has now enabled precise genetic manipulation across diverse mushroom species, such as *A. bisporus* (Choi et al., 2023a), *Ganoderma lucidum* (Eom et al., 2023; Liu et al., 2020; Qin et al., 2017; Wang et al., 2020) *Lentinula edodes* (Moon et al., 2021), and *Pleurotus ostreatus* (Boontawon et al., 2021a; Kobukata et al., 2024; Koshi et al., 2022; Yamasaki et al., 2021). However, a persistent challenge in mushroom genetic manipulation lies in the restoration of dikaryotic states post successful gene editing. This necessitates editing alleles in two monokaryotic strains of compatible mating types to assess the editing outcomes in fruiting bodies. Fruiting bodies mainly develop from dikaryotic mycelia formed by mating two monokaryotic strains of compatible mating types. For example, the effect of *PPO1* deletion in the fruiting bodies of *A. bisporus* was demonstrated using a dikaryotic mycelia made by mating a wild-type monokaryon and a *PPO1*-edited monokaryon (Choi et al., 2023a). Genes, such as *mer3*, *msh4*, *pcl1*, and *cro6c*, related to basidiospore production in *P. ostreatus* were investigated through the mating of gene-edited monokaryons (Kobukata et al., 2024; Yamasaki et al., 2021). Recent reports have described the editing of both nuclei using plasmid-based CRISPR/Cas9 technology in *P. ostreatus* (Kobukata et al., 2024; Yamasaki et al., 2022).

Gene editing in mushrooms has been approached using two main methods: delivery of plasmid DNA encoding Cas9 and guide RNA (gRNA), or directly introducing the Cas9-gRNA ribonucleoprotein complex (RNP) into the cytoplasm of mycelial cells. Plasmid DNA transformation typically involves polyethylene glycol (PEG), which creates transient channels in the cell membrane (Boontawon et al., 2021a; Koshi et al., 2022; Liu et al., 2020; Qin et al., 2017; Wang et al., 2020; Yamasaki et al., 2021). Alternatively, transformation has been achieved by infecting mycelia with *Agrobacterium tumefaciens* carrying the desired plasmid (Choi et al., 2023a; Zhang et al., 2022). These plasmid DNA-based approaches introduce complexities related to the transcription and translation of Cas9 and the transcription of gRNA within the host cytoplasm. Direct introduction of RNP to the cytoplasm has also been effective for gene editing in filamentous fungi (Boontawon et al., 2021b; Ceccaldi et al., 2016; Eom et al., 2023; Pareek et al., 2022; Vonk et al., 2019). This method is advantageous over plasmid DNA-based gene editing systems since it bypasses the needs for host factors involved in Cas9 and gRNA production.

In this study, we assessed gene editing of both nuclei in a dikaryotic strain of *G. lucidum* using RNP targeting *pyrG*. Disrupting *pyrG* allows for positive selection on a 5-fluoroorotic acid (5-FOA)-containing medium. The nuclear type (karyotype) of the transformants was determined by the investigation of the *A* mating type locus, as effectively applied for *L. edodes* (Ha et al., 2018) and *A. bisporus* (Choi et al., 2023b). The disruption of *pyrG* in each nucleus was confirmed by sequencing the target region. We found that the edited nuclei were stably maintained through serial transfers. Finally, we evaluated the fruiting characteristics of the edited dikaryon.

## Materials and Methods

### Strain and culture conditions

*G. lucidum* GL3315 collected from Sorak mountain was obtained from the wild mushroom stock center (Prof. T. S. Lee, Incheon National University). The mushroom strain was cultured on YPDA medium composed of potato dextrose agar (PDA; Oxoid, UK) and yeast extract (5 g/L) at 28℃. YMGUU+FOA medium composed of 4 g/L of yeast extract, 10 g/L of malt extract, 4 g/L of glucose, 20 mg/L of uracil, 5 g/L of uridine, 1 g/L of 5-fluoroorotic acid (5-FOA), and 1.5% agar was employed for the selection of the *pyrG*-disrupted transformants.

### Generation of monokaryotic strains by de-dikaryotization

Monokaryotic strains, M1 and M2, of GL3315 were generated by de-dikaryotization as previously described (Ha et al., 2019). In brief, mycelia of GL3315 grown in PDB (Oxoid, UK) were collected and washed twice with sterile deionized water. The mycelia were subjected to the cell wall lysing enzyme treatment for 4 h at 30℃ with β-glucanase (0.1 g; Amicogen, Korea), chitinase (0.04 g, Chitimax-N; Amicogen, Korea), and cellulase (0.1 g; Sigma-Aldrich, USA) in 10 ml of 0.5 M sorbitol. Resulting protoplasts were collected by filtration with Miracloth (Merck, Germany). The filtrate was spread on PDA containing 0.5 M sorbitol. Mycelial colonies grown on PDA were isolated to new PDA to determine their karyotypes.

### Karyotyping

**Fig. 1.**
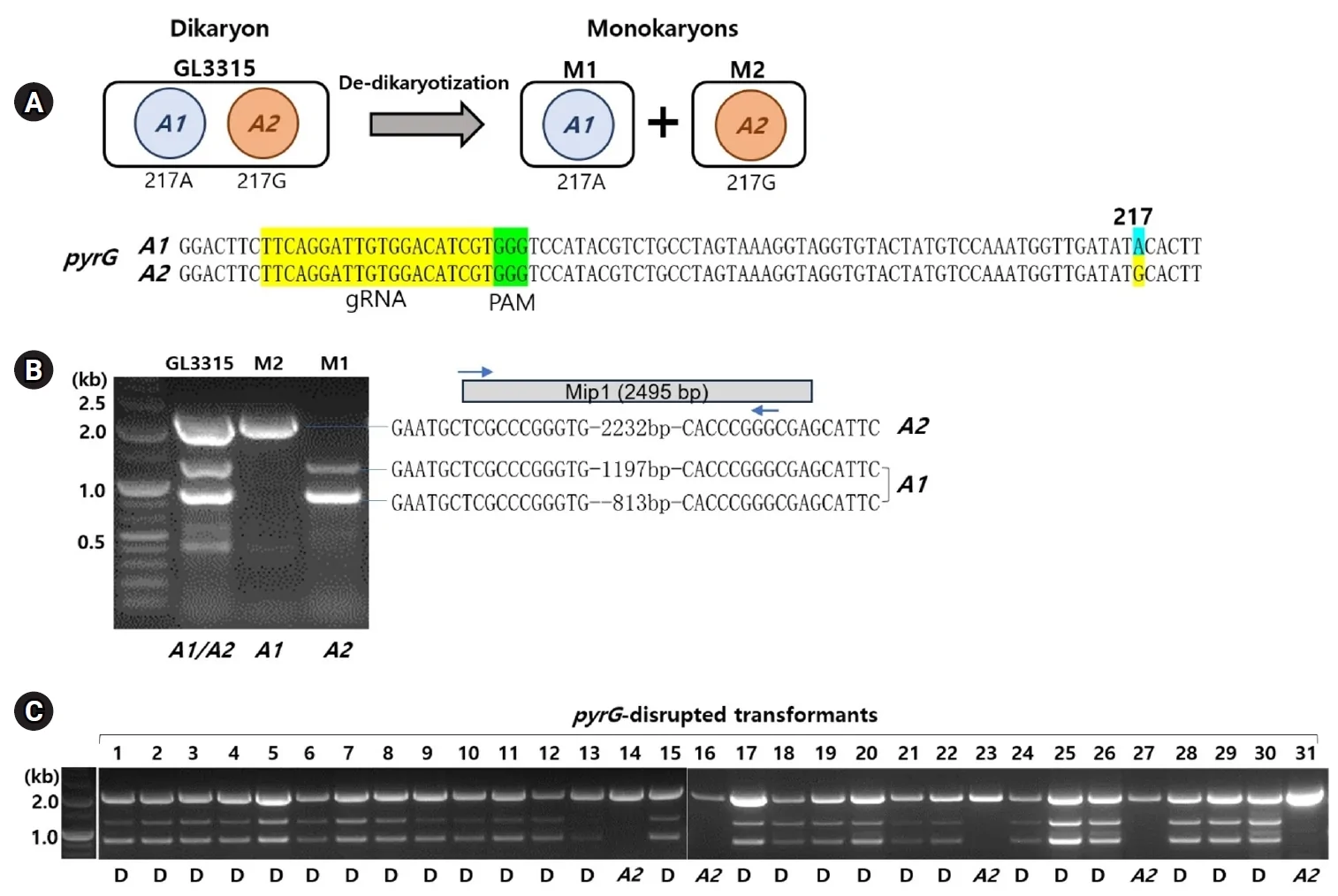
Identification of karyotypes in *Ganoderma lucidum* GL3315. (A) Monokaryotic strains M1 and M2, derived from the dikaryotic GL3315 strain through de-dikaryotization, each possess one of the two nuclei from the original dikaryotic strain. Sequence analysis of the *pyrG* gene in these monokaryotic strains revealed a single-nucleotide polymorphism (SNP) at position 217: strain M1 has an 'A' at this position (designated SNP^217A^), while strain M2 has a 'G' (designated SNP217G). The gRNA target sequence within *pyrG* is highlighted in yellow, and the protospacer adjacent motif (PAM) is highlighted in green. Notably, SNP217 is located near the PAM sequence. (B) Karyotyping was performed using MIP1 sequence variation. PCR amplification of the MIP1 gene produced 1,231 bp and 847 bp amplicons from the M1 strain, and a 2,272 bp fragment from the M2 strain. The PCR amplicons from both strains contain identical inverted repeat sequences. (C) Karyotyping of transformants survived on YMGUU+FOA medium. 'D' under the gel image denotes dikaryotic strains, while 'A2' indicates a monokaryotic strain with the *A2* karyotype.

Determination of karyotypes of M1, M2, and *pyrG*-disrupted isolates was approached by the investigating clamp connections under a light microscope, PCR targeting the *MIP1* gene using a specific primer set (Table S1), and identification of a single-nucleotide polymorphism (SNP) in *pyrG* located at 217th nucleotide from the start codon (Fig. 1). Four *MIP1* sequences downloaded from Mycocosm (https://mycocosm.jgi.doe.gov/mycocosm) were compared to derive consensus sequences for primer design (Supplementary data S1, Table S1). PCR was conducted under the following conditions: initial denaturation at 95°C for 5 min, followed by 25 cycles of denaturation at 95°C for 30 sec, annealing at 60°C for 30 sec, extension at 72°C for 2 min, and a final extension step at 72°C for 5 min.

### Disruption of *pyrG* by RNP

Cas9 and gRNA targeting the *pyrG* gene were prepared according to the protocol outlined by Eom et al. (2023) and utilized for transforming GL3315. In summary, Cas9 (10 µg) and gRNA (5 µg) were combined in 20 µl of assembly buffer (50 mM Tris-HCl, 0.1 M NaCl, 10 mM MgCl2, 1 mM DTT, pH 7.9) and incubated at 37°C for 30 min. The resulting RNP solution was mixed with a protoplast solution containing 10^7^ protoplasts in 0.2 ml of STC buffer (10 mM Tris-HCl, 10 mM CaCl_2_, 1 M sorbitol, pH 7.4), supplemented with 2 µl of 0.6% Triton X-100, and kept on ice for 40 min. Following this, 1 ml of PTC buffer (STC buffer with 40% PEG4000 and 10 µl of 0.6% Triton X-100) was added, and the reaction mixture was further incubated for 20 min at 25°C. The transformed mixture was plated onto YMGUU+FOA medium containing 0.5 M sorbitol for subsequent screening of transformants.

### Analysis of the transformants

Disruption of *pyrG* in the transformants was analyzed by PCR and subsequent sequencing. The complete sequence region of *pyrG* (1.1 kb) was amplified using a primer set (Table S1) and analyzed by agarose gel electrophoresis. The DNA fragments in the gel were extracted and cloned into TOPcloner TA plasmid (Enzynomics, Korea) for the sequence determination.

### Fruiting body production

Mycelial suspension (20 ml) grown in PDB was inoculated to sawdust substrate, containing 190 g of sawdust, 10 g of rice bran, 5 g of crushed oyster shell, and 300 ml of water, in a polypropylene bottle (500 ml). The inoculated bottles were incubated at 25°C for 30 days in the dark. Fruiting was induced by scratching top mycelial layer of the fully developed mycelial bottle. The scratched bottles were incubated at 30°C for 30 days under white light.

## Results

### Karyotyping of the transformants

De-dikaryotization of the dikaryotic GL3315 yielded two monokaryotic strains, M1 and M2. The monokaryotic nature of M1 and M2 was confirmed by the absence of clamp connections in their growing mycelia, which are characteristics of dikaryotic mycelia. The karyotypes of M1 and M2 were assessed by PCR targeting *MIP1*, a component of the *A* mating type locus. For this assessment, we compared four *MIP1* sequences of *Ganoderma* species obtained from public databases to identify consensus sequences (Supplementary data S1). PCR amplification using primer sets derived from these consensus sequences produced amplicons of 1,231 bp and 847 bp from the M1 strain and a 2,272 bp fragment from the M2 strain. Sequence analysis of the 2,272-bp fragment confirmed it as the *MIP1* gene for the M2 strain, amplified by the forward primer binding to the 5’-sequence region of *MIP1* and the complementary 3’-sequence (Fig. 1B). Analysis of the 1,231-bp and 847-bp amplicons from the M1 strain revealed that they corresponded to a long terminal repeat (LTR) sequence and a gene encoding a histone acetylation domain-containing protein, respectively, both of which have inverted repeats at their ends (Fig. 1B, Supplementary data S2). This suggests the involvement of LTR-retrotransposons in the diversification of the *A* mating type locus, similar to observations in *Agaricus bisporus* (Choi et al., 2023b). By analyzing the presence of nucleus-specific PCR bands, we were able to differentiate the karyotypes of M1 and M2 as *A1* and *A2*, respectively (Fig. 1). This karyotyping was further validated by the identification of a single-nucleotide polymorphism (SNP) at the 217^th^ position of *pyrG*, where *A1* exhibited an ‘A’ (SNP^217A^) and *A2* exhibited a ‘G’ (SNP^217G^) (Fig. 1A).

### Analysis of *pyrG*-disrupted transformants

**Fig. 2.**
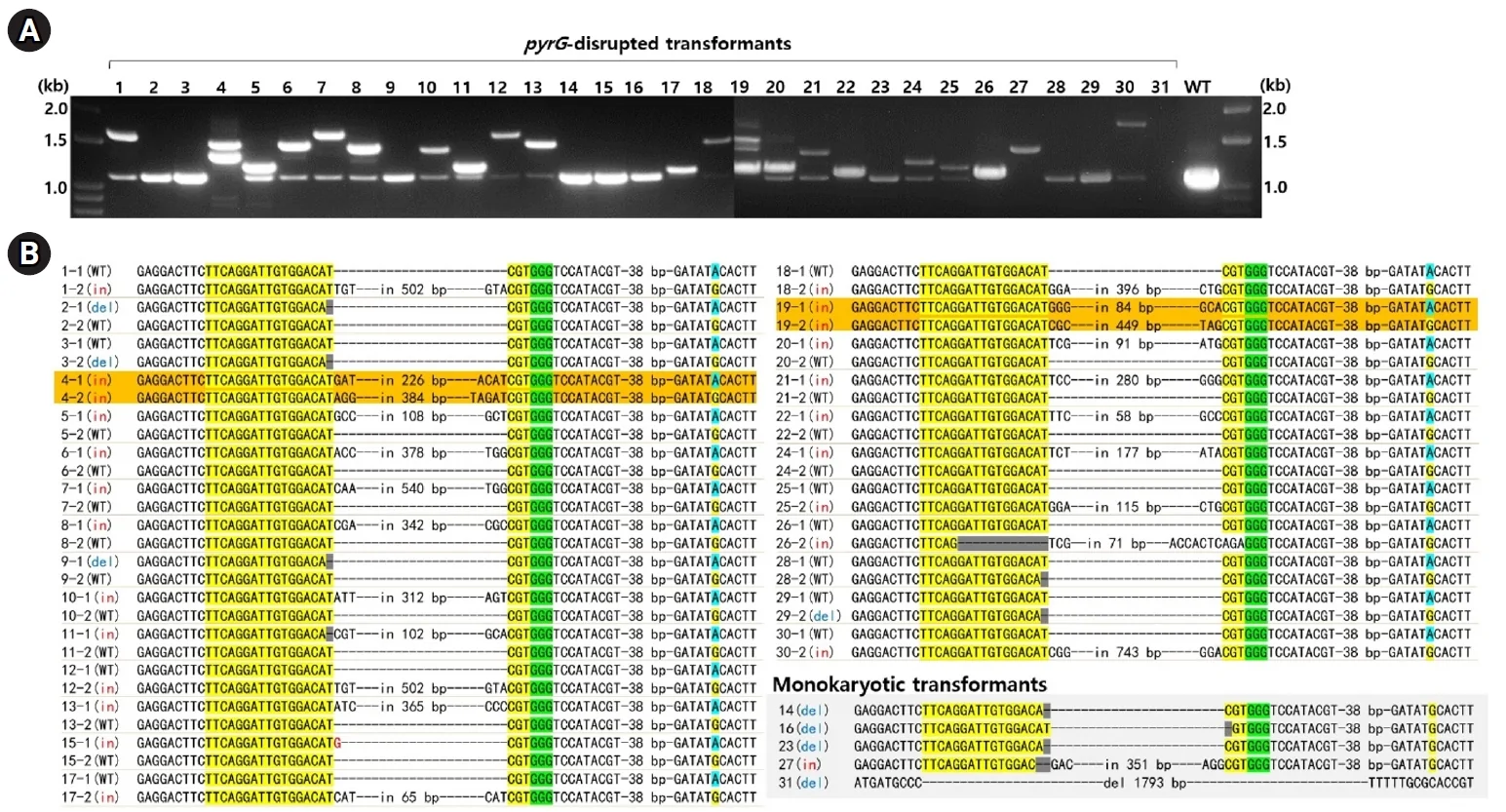
Analysis of *pyrG*-disrupted transformants. (A) PCR amplification using a primer set targeting the *pyrG* gene revealed that the undisturbed *pyrG* produced a 1.1 kb amplicon. In contrast, pyrG gene disruptions resulted in amplicons of varying sizes. (B) Sequence analysis of the PCR amplicons was conducted following TA cloning. The sequences from the *pyrG* gene surrounding the RNP-mediated DSB sites are presented here. The crRNA target sequences are highlighted in yellow, with PAM indicated in green. Dikaryotic strains with disruptions in both nuclei are marked in orange. Detailed sequence information for the insert DNA is available in Table S2. The SNP217, which represents the karyotype, is highlighted in sky blue or yellow.

The treatment with RNP and subsequent selection on 5-FOA (1 g/L) yielded 31 transformants, which survived on fresh YMGUU+FOA medium. Karyotyping of these transformants using the primer sets specific to the *A* mating type loci revealed that most dikaryons, indicated by the presence of both *A1* and *A2* mating type markers (Fig. 1C). This was further confirmed by the concurrent presence of SNP^217A^ and SNP^217G^ (Table 1, Fig. 2B). Only five of the transformants, including N14, N16, N23, N27, and N31, were monokaryotic, containing the *A2* nucleus with SNP^217G^ (Figs. 1C and 2B, Table 1).

The disruption of *pyrG* in the transformants was analyzed by PCR using primer set targeting the entire *pyrG* sequence region. PCR amplification showed a 1.1-kb DNA band in most of the transformants, while some exhibited additional bands of various sizes (Fig. 2A). Sequencing of the amplicons revealed that 10 transformants carried the *A1* nucleus with the wild-type (*pyrG*+) and the *A2* nucleus with disrupted *pyrG* (*pyrG*-) whereas 14 transformants carried *pyrG*- in the *A1* nucleus and *pyrG*+ in the *A2* nucleus (Fig. 2B, Table 1). The *pyrG* disruption was caused by a single base deletion or insertions, along with variable-sized fragments of chromosomal or mitochondrial DNA (mtDNA) (Fig. 2B, Table S2). Transformants N4 and N19 were the only two ones with disrupted *pyrG* in both nuclei (Fig. 2B). In these transformants, the disruption was facilitated by the insertion of fragmented chromosomal DNA or mtDNA (Table S2).

### Stability of the edited nuclei

**Fig. 3.**
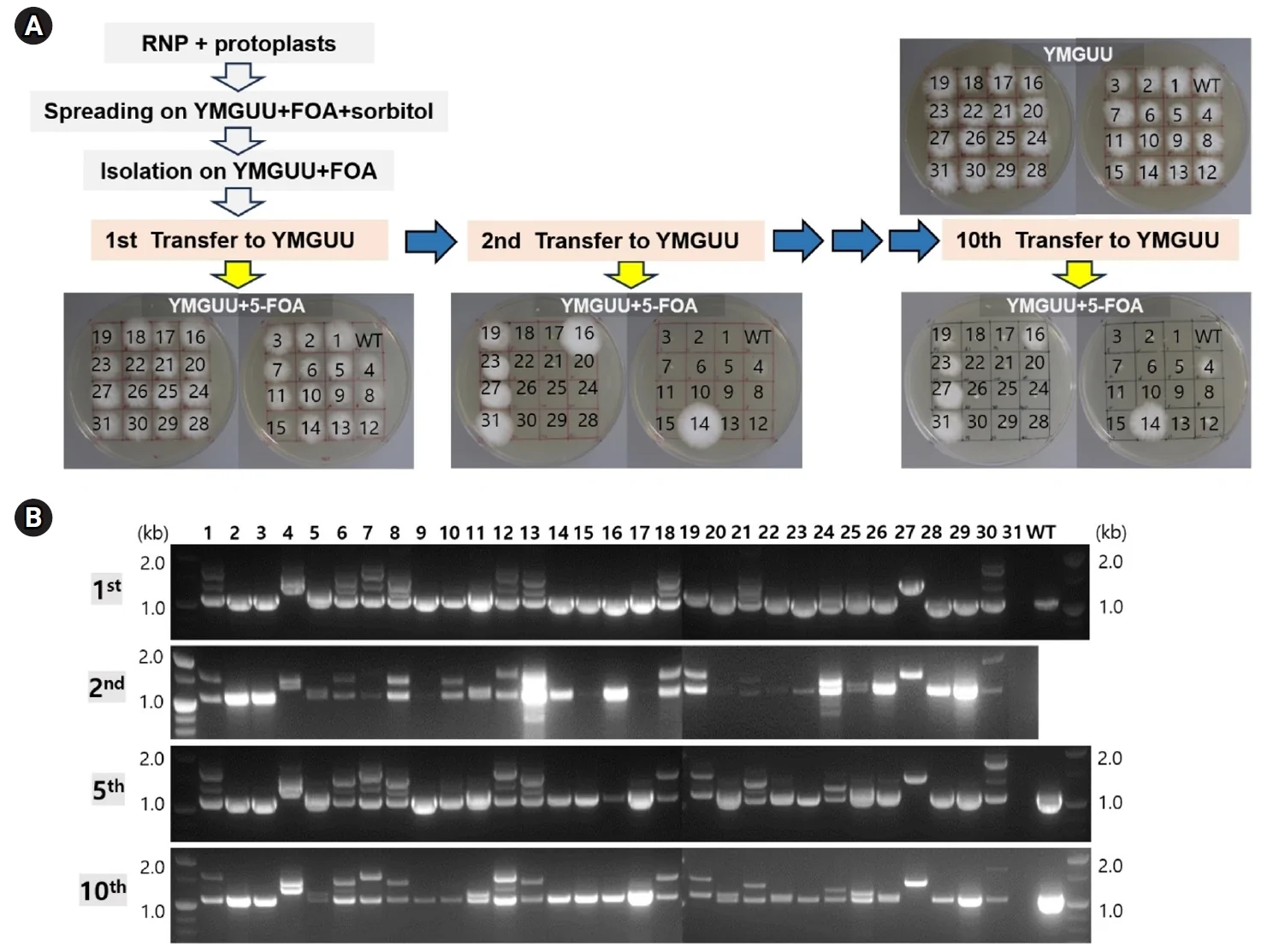
Genetic stability of the transformants. (A) The transformants isolated from YMGUU+FOA were first transferred to YMGUU medium (1st transfer). Mycelial blocks from this initial transfer were subsequently evaluated on YMGUU+FOA and concurrently transferred to fresh YMGUU medium (2nd transfer). This process was repeated through a series of 10 consecutive transfers, with each transfer involving a 2-week incubation period. (B) PCR analysis of the *pyrG* gene was performed on colonies from selected transfers using colony PCR. The results of the PCR analyses were consistent across the different transfers.

**Fig. 4.**
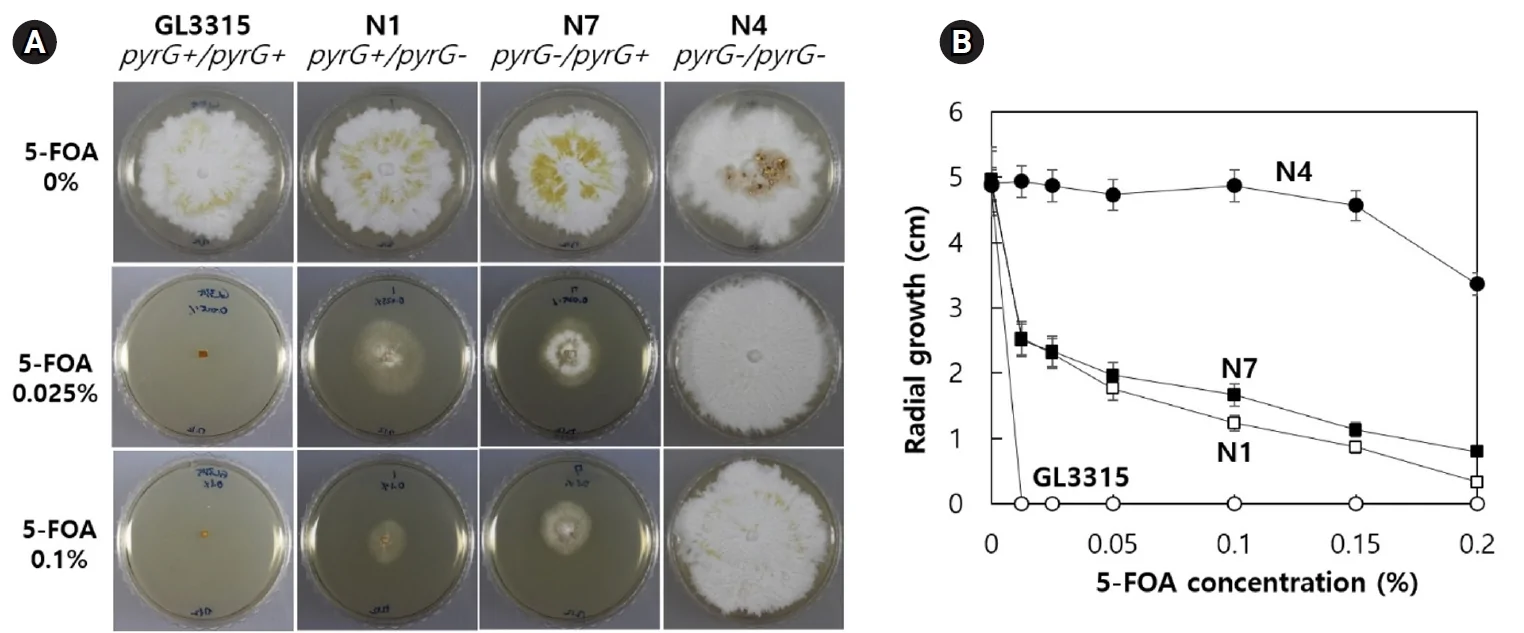
Resistance of the transformants having different genotypes against 5-FOA. (A) Resistance of the *pyrG*-disrupted transformants on YMGUU containing different concentrations of 5-FOA. (B) Effect of 5-FOA concentration on the growth of the transformants. Radial growth, presented from triplicate measurements, show the impact of different 5-FOA concentrations on *pyrG* disruption.

The stable maintenance of gene-edited transformants was evaluated through serial transfers onto YMGUU medium without selective pressure. Mycelial colonies propagated on YMGUU+FOA supplemented with 0.5 M sorbitol were initially isolated and transferred to fresh YMGUU+FOA medium. Subsequently, these isolates were further transferred to YMGUU medium to establish 31 gene-edited transformants (Fig. 3A, 1st transfer). In a growth assay on YMGUU+FOA, all transformants exhibited significant growth, unlike the wild-type GL3315 strain. However, heterokaryotic transformants with an intact nucleus (*pyrG*+/*pyrG*- or *pyrG*-/*pyrG*+) showed highly retarded growth on YMGUU+FOA from the 2nd transfer onwards (Figs. 3 and 4), due to the conversion of 5-FOA to the toxic 5-fluorouracil (5-FU) by the wild-type *pyrG* gene product. Only transformants with disruption of *pyrG* in both nuclei (*pyrG*-/*pyrG*-) of dikaryotic strains (N4 and N19) and *pyrG*-disrupted monokaryotic strains (N14, N16, N23, N27, and N31) showed a normal growth on YMGUU+FOA. Despite the lethality of 5-FOA, PCR analysis of the *pyrG* sequence regions in the transformants which were serially transferred to YMGUU indicated that the nuclei were stably maintained (Fig. 3B).

Selected transformants representing each genotype were exposed to reduced concentrations of 5-FOA to investigate the effects of single or double disruption of *pyrG* in dikaryotic strains. The double-disruption mutant N4 strain (*pyrG*-/*pyrG*-) exhibited high resistant to 5-FOA, showing resistance to concentrations higher than 0.2% 5-FOA, whereas single disruption transformants N1 (*pyrG*+/*pyrG*-) and N7 (*pyrG*-/*pyrG*+) showed a retarded growth on YMGUU containing more than 0.0125% 5-FOA (Fig. 4). In contrast, the wild-type GL3315 was sensitive to 5-FOA greater than 0.005% (data not shown).

### Fruiting characteristics

**Fig. 5.**
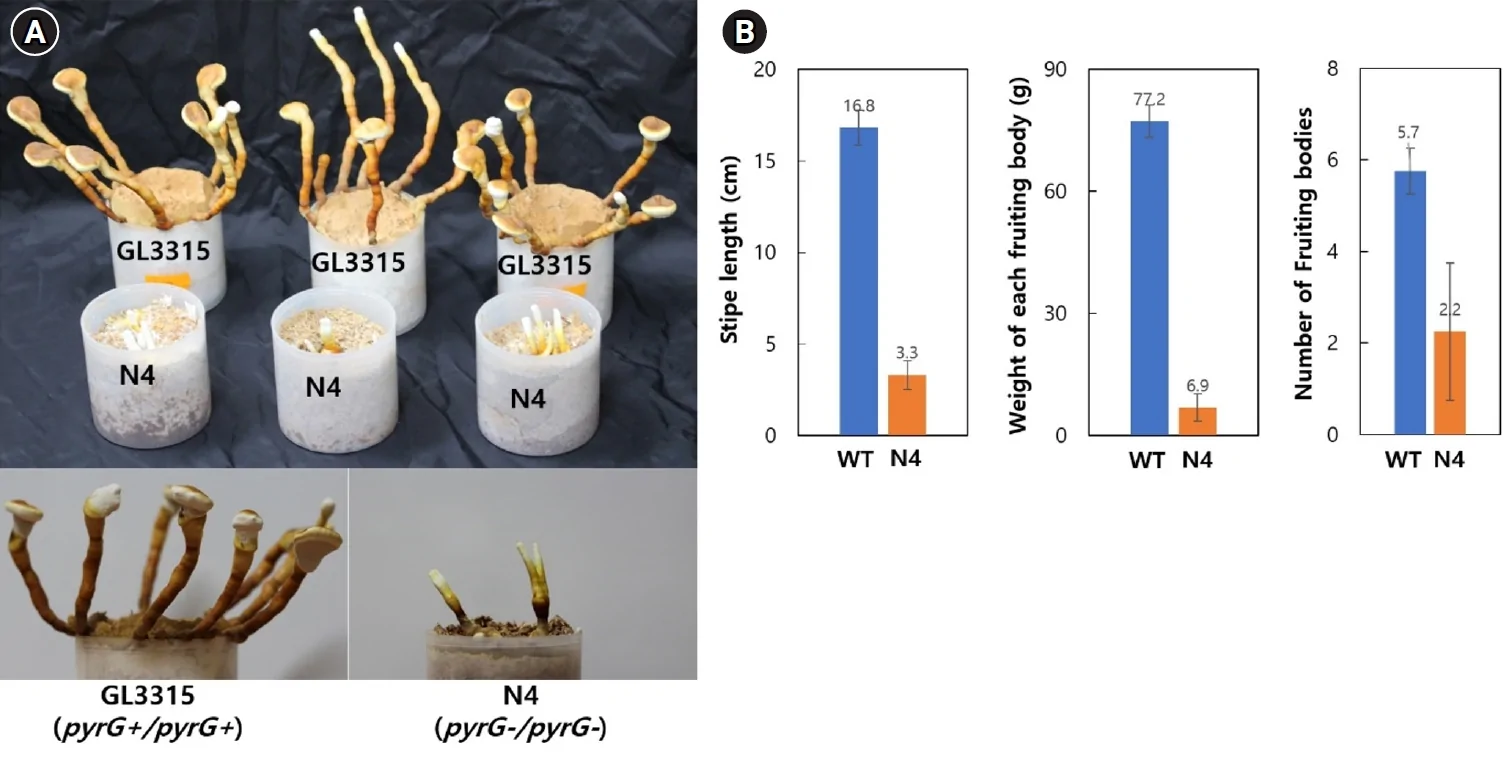
Fruiting characteristics. (A) Fruiting bodies were produced on a sawdust substrate. The mycelia were cultured for one month, followed by an additional month of incubation after fruiting was induced by physical scratching. (B) Characteristics of fruiting bodies from the wild-type GL3315 and the double disrupted N4 strain. The data represent the average values obtained from fruiting bodies collected from three separate substrate bottles.

Fruiting bodies of strains GL3315 (*pyrG*+/*pyrG*+) and N4 (*pyrG*-/*pyrG*-) were cultivated on sawdust substrate in a growth chamber maintained at 30°C with 85% relative humidity without aeration. Cultivation at this condition produced fruiting bodies of elongated stipes with small pilei (Fig. 5A). On average, GL3315 produced 5.7 fruiting bodies per substrate bottle, weighing 77.2 g each (Fig. 5B). In contrast, N4 produced only 2.2 fruiting bodies per bottle, with an average weight of 3.3 g. Additionally, the average stipe length was 16.8 cm for GL3315, whereas N4 exhibited shorter stipe length of 3.3 cm. Notably, N4 did not develop pileus and consequently failed to produce basidiospores.

## Discussion

Gene editing in fungi has been impeded by difficulties associated with the expression of Cas9 and guide RNA (gRNA) within the mycelial cytoplasm. These challenges are further complicated by heterokaryosis, the presence of multiple nuclei within a single cytoplasm, which is distinct from plants and animals, where typically only a single diploid nucleus is present. Fungal gene editing generally affects one of the multiple nuclei, as seen in cases like (pyrG-/pyrG+) or (pyrG+/pyrG-) in this paper, because Cas9-gRNA is more likely to enter one nucleus rather than both simultaneously. In this study, we demonstrate that these challenges can be mitigated by directly introducing RNPs into protoplasts. Furthermore, our investigation into mating-type genes provides evidence of our ability to differentiate between nuclear types in dikaryotic cells and precisely identify gene-edited nuclei.

Through RNP transformation of dikaryotic protoplasts, we obtained 31 transformants, a number comparable to our previous findings with monokaryotic strains (Eom et al., 2023). Analysis of *pyrG* gene disruptions among these transformants revealed two instances where *pyrG* was disrupted in both nuclei, underscoring the difficulty of simultaneous editing in both nuclei of a dikaryon. Interestingly, within these transformants, five were monokaryotic, presumably resulting from de-dikaryotization during the regeneration process (Ha et al., 2019). The remaining 24 transformants harbored the edited *pyrG* gene only one of the two nuclei: 10 transformants exhibited the *A1* nucleus with *pyrG*+ and the *A2* nucleus with *pyrG*-, while 14 transformants showed the opposite configuration with the *pyrG*- *A1* nucleus and the *pyrG*+ *A2* nucleus. Initially, these transformants exhibited resistance to 5-FOA following the first transfer to YMGUU medium. However, this resistance decreased after subsequent transfers to fresh YMGUU medium (Fig. 3A). Prolonged incubation of *pyrG*+/*pyrG*- or *pyrG*-/*pyrG*+ mutant strains on individual YMGUU+FOA (0.1%) plates revealed that these strains grew more slowly compared to the *pyrG*-/*pyrG*- strain (Fig. 4). This suggests that the presence of a single copy of *pyrG*+ in one of the two nuclei confers insufficient toxicity due to reduced conversion of 5-FOA to the lethal compound 5-FU.

The observed growth pattern, where resistance is only apparent in the first transfer, may be due to a biased proportion of nuclei with *pyrG*+ versus *pyrG*-. Mushroom mycelial cells often harbor multiple nuclei with varying proportions of two nuclear types depending on developmental stages and environmental conditions (Gehrmann et al., 2018). Selection by two consecutive transfers on 5-FOA may result in a higher proportion of transformants retaining the *pyrG*- nucleus compared to the *pyrG*+ nucleus. This proportion could be disrupted when transferred to fresh medium without selective pressure, as the initial transfer serves as a transitional phase to establish a new nuclear composition, which could increase the number of *pyrG*+ nuclei. Nucleus-specific expression of a gene observed in *A. bisporus* (Gehrmann et al., 2018) and *L. edodes* (Ha et al., 2017) is unlikely in this instance, as the loss of resistance, a marker of *pyrG*+ activity, occurs without discriminating the origin of *pyrG*+, whether it is from the *A1* nucleus or from the *A2* nucleus.

We demonstrated that fruiting in N4 (*pyrG*-/*pyrG*-) was significantly impaired, with the formation of the pileus, which is essential for spore production, also being disrupted. This inhibition is due to the loss of *pyrG* function, which interferes with pyrimidine nucleotide biosynthesis. Consequently, the necessary pyrimidine nucleotides must be obtained from the medium, as the sawdust substrate used for vegetative growth does not provide an adequate supply of these nucleotides for fruiting body development.

In summary, our study addresses key challenges in gene editing within mushroom systems by employing RNP complexes directly in protoplasts. Our findings reveal that while RNP transformation of dikaryotic protoplasts yielded a comparable number of transformants to monokaryotic strains, achieving simultaneous gene editing in both nuclei of a dikaryon remains challenging. Notably, only one of the two nuclei in most transformants harbored the edited *pyrG* gene, with variable configurations observed between the *A1* and *A2* nuclei. Additionally, the impairment of fruiting in N4 (*pyrG*-/*pyrG*-) suggests the critical role of *pyrG* in pyrimidine nucleotide biosynthesis. These insights contribute to a deeper understanding of gene editing in fungi and suggest potential avenues for overcoming the inherent difficulties associated with heterokaryosis.

## Figures and Tables

**Table 1.** Analysis of *pyrG*-disrupted transformants

Transformant No.	Karyotyping			*pyrG* in nuclei		Genotype (A1/A2)
	Clamp	Nuclear type	SNP217	*A1* nucleus	*A2* nucleus	
1	O	*A1/A2*	A/G	WT	insertion 502	*pyrG+*/*pyrG-*
3	O	*A1/A2*	A/G	WT	deletion 1	
12	O	*A1/A2*	A/G	WT	insertion 502	
17	O	*A1/A2*	A/G	WT	insertion 65	
18	O	*A1/A2*	A/G	WT	insertion 396	
29	O	*A1/A2*	A/G	WT	deletion 1	
30	O	*A1/A2*	A/G	WT	insertion 743	
25	O	*A1/A2*	A/G	WT	insertion 115	
26	O	*A1/A2*	A/G	WT	insertion 71	
28	O	*A1/A2*	A/G	WT	deletion 1	
2	O	*A1/A2*	A/G	deletion 1	WT	*pyrG-/pyrG+*
5	O	*A1/A2*	A/G	insertion 108	WT	
6	O	*A1/A2*	A/G	insertion 378	WT	
7	O	*A1/A2*	A/G	insertion 540	WT	
8	O	*A1/A2*	A/G	insertion 342	WT	
9	O	*A1/A2*	A/G	deletion 1	WT	
10	O	*A1/A2*	A/G	insertion 312	WT	
11	O	*A1/A2*	A/G	insertion 102	WT	
13	O	*A1/A2*	A/G	insertion 365	WT	
15	O	*A1/A2*	A/G	insertion 1	WT	
20	O	*A1/A2*	A/G	insertion 91	WT	
21	O	*A1/A2*	A/G	insertion 280	WT	
22	O	*A1/A2*	A/G	insertion 58	WT	
24	O	*A1/A2*	A/G	insertion 177	WT	
4	O	*A1/A2*	A/G	insertion 226	insertion 384	*pyrG-/pyrG-*
19	O	*A1/A2*	A/G	insertion 84	insertion 449	
14	x	*A2*	G	X	deletion 1	*pyrG-*
16	x	*A2*	G	X	deletion 1	
23	x	*A2*	G	X	deletion 1	
27	x	*A2*	G	X	insertion 351	
31	x	*A2*	G	X	deletion 1793	

## Data Availability

No data was used for the research described in the article.

## Supplementary Information

Table S1. Primers in this study

Table S2. Origins of the insert DNAs

Supplementary data S1. *MIP1* sequences of *Ganoderma* species

Supplementary data S2. Sequences of PCR amplicons
