# Supplementary material for: Simultaneous gene editing of both nuclei in a dikaryotic strain of Ganoderma lucidum using Cas9-gRNA ribonucleoprotein
Source: J Microbiol. 2025 Jan 24;63(1):e.2409006. doi: 10.71150/jm.2409006 (PMC13577092; doi:10.71150/jm.2409006)
Supplement: Supplementary file 1 [file jm-2409006-Supplementary-Table-S1.pdf]

Supplementary data

Table S1. Primers in this study

Primer name	Sequence (5' - 3')	Remark
G.L_pyrG_seq_F	GCAAAACTGGAAAACAGCGCG	pyrG gene sequencing
G.L_pyrG_seq_R	ACTGTGCTACTATACCCGTCTC	
G.L_pyrG_gRNA2	TAATACGACTCACTATAGGTTCAGGATTGTGGACATCGTGTTTTAGAGCTAGAAATAGCAAGT	synthesis gRNA2 of pyrG
gRNA-R	AAAAAAgcaccgactcggtgcca	
G.L_mip-F	GAATGCTCGCCCGGGTG	mip sequencing
G.L_mip-R	GATGGCACGATCAAAGAGGTAGG	
G.L_pyrG_Up_F	GCGAGCGTCTTCAAATGGATG	Tf31 pyrG sequencing
G.L_pyrG_Dn_R	CAGGTTCTTCAAGATGCTAGCC	

Table S2. Origins of the insert DNAs

No.	Inserted DNA size (bp)	Origin of the insert DNA	Inserted DNA Sequence
**1**	502	*9:1187485-1187628, 30:129758-129896 12:826662-826766, 7:1965253-1965345	TGTTATAGCCGGGTCGTGCTTGTAGCCGGGTCGCATGGGCGGCATAACTAAACTCGGGCGGGTATGCAGCGTTCATCACTAGTACTCTCAGGCGATCTTGCCCGGTTTCTCGTCCGACGTGTTCGCGTGGGCCAATCATGCGCCTCTCTGTCTTATTCCTGTCCCCCTCCCCCTCATCTCGACTTCTCCCTGCTCTCGTCGCTGCCTGACGGGCAGGCCAGCCCATGCAGTTCGCGTACAGAGTAATTCACTCTGGCTCTACGACTGCACCAATGGGTACCATCTGGATATGACTCTCGTGCACGCGCGGGGCGTCCGGGGCGTCGGCTGCGGCGGCGACTGAGGAGGGGAAGGAGAACACGCCGGAGCCGGGGGCGGACGAGGAGAATGAGAAAGAGGAGGCAACTTCGACATTTCCCTAGCGTCGTCTAACGAGTAGTGAGTCATGTGTGCAGACTATCTCGTAAACGTTTACTCACGTCCCATGCCTCTGCAGCGCGTA
**5**	108	21:699229-699331	GCCGATCATTGAGATGCCTGTCCCTTGTCTTGACCACCCACTGCACAGTATTCTTGAAATCCAACATGAGCCTCTGGCCGACAACCATCGATTTCCGCGTTCGGAGCT
**6**	378	5:1270291-1520218, mtDNA, 14:646678-646679	ACCCGGTGTACAAAGGCTACTATGAACTCCCTGACATGATCAAGGCCAAGGGCTCAGCATCTGGCACTCGACTACGTGCTATGGACGAAGAAAATTATTATAAACTTTGATTGTTTTTATTTGTTCTACAATTTTACTTAAAAAACAAGTTGAGTATTCGATATCAAAATATGGAAAAGACTCGTACCAGCGCACCCACTCCTGTGTTGCCACCAAGGAAATGATCGGAAAGCGTTAAATCTAGGGATAGTCACAGGCCTATGTGGATGTCGAATTGACAGGGTTCAGAAATGTTGTCGATTTTGAATTGCGAAAGCCTTGCAACCATCTGGGGACACTGACAAGAGGAAACCATTTAGGGAATACGTTTTTGGTTGG
**7**	540	32:54644-54812, 6:1597390-1599980 15:775042-774945, 17:497829-497928 4:1550725-1550633, 8:1500509-1500539	CAACGAGCTGCTCGCGCGCCTGACGGGCCAGCCGGTGCGCGACCACACGCAGACGAACTCGACGCTGGAGATGCATACAAGCAAGAGGACGAGGCAGCCGAGGTTCGTGAATCCTCGCATGGAGAACACGTGACCGTTTCCCTTCCCCTTCTCCTTCGTAGTATCCCCCCGACTCCCCTTCATCCCACTCGTCCTCCTCTTCTTCGCCCTGAAGGACATCTCCAAGGGCCAAAAGTAAACTTGTCGTGCGGGGAGACCGGATCGTCAGTACTTCGTCCTGACTTGAGTCCCAAATCCAAGCCCCCGTCATCCGATCGTCCGCCTGCCAATCCAGCCCCATCTCGCTCAGAAAGTCAATATGTTGGAATAGGGGTGTTACTCACTGCGAAACTGTTGTCGCACTTTCTCCGGCTGCAGCCAAACGGGCCCAGGGACTCCAAAGGCAGGTGGTACGAAGCCCGTCTCCCGCCCACCTCGCCACTTCGATGAGGAGCTCGCGTTTCTTGTCGACACCTGCCATCGAACGGAGCTCATAGAGCG
**8**	342	unidentified	CGATCGCTCACCCTTAGAATTAACACGTCGAGATGGCGTCCCCTCGCACCTCCGACTCAGTAAAGAACTCTCGCCCTGTCGACGCGCGAAAGTCCCAATGCCGCACACCCGCCATAAACCCAGCCAAGGCCACGAAGCTAGCAGATCCAGCCGCGAAGGCTGCTGAGCAGGCACAGACCGAGGAGGCGTCGGACTACGGCAGCGACACCTACGTTGAGATAGACAACACTGAGCGGCTTCCTGCGAAGAAGGCCAAGACAATGCCGCCAGCGCGCCGGTCGTCCTACAATGTCATCGCCGGTTGCATTCTGTCCCACGCCGAACCGATTGCTCGGTGCTCGC
**10**	312	mtDNA	ATTACCTTCTTTAGGTTTAATTAAATTTTGAGAATAGCTTAATTTAGCTTCTAATATTTCATCGCTATTTATTCTATAGAAAAATATTAATTCTTTAATAATAAAATCTCTATTATCAAAATATTAGGATACTCATTTCCCACTAAATTAAATTTAGATAGCTGGGGAATGGATATAATTTCGTATATTTTTTTCTTATTATGTCCTGTCTAAGATCATCTTTAATTATATTAATTTTTTCTATTACTTTTGATAGGAGAGTGTCTGTTTGATGAGTTAAATCTTTGGCCACATGACTTTTATCTTGATAGT
**11**	102	8:1428154-1428255	GGTCAGACCGATCTTGAGCGGGATGCGGAAGCCAGTCGCGCGGCGGACGGGAGTCCAGCCGGCAGGGACGAAGCTGCGAGACTCGTGGAGGACCCGGGGCGA
**12**	502	9:1187485-1187628, 12:826766-826662, 30:129758-129896	TGTTATAGCCGGGTCGTGCTTGTAGCCGGGTCGCATGGGCGGCATAACTAAACTCGGGCGGGTATGCAGCGTTCATCACTAGTACTCTCAGGCGATCTTGCCCGGTTTCTCGTCCGACGTGTTCGCGTGGGCCAATCATGCGCCTCTCTGTCTTATTCCTGTCCCCCTCCCCCTCATCTCGACTTCTCCCTGCTCTCGTCGCTGCCTGACGGGCAGGCCAGCCCATGCAGTTCGCGTACAGAGTAATTCACTCTGGCTCTACGACTGCACCAATGGGTACCATCTGGATATGACTCTCGTGCACGCGCGGGGCGTCCGGGGCGTCGGCTGCGGCGGCGACTGAGGAGGGGAAGGAGAACACGCCGGAGCCGGGGGCGGACGAGGAGAATGAGAAAGAGGAGGCAACTTCGACATTTCCCTAGCGTCGTCTAACGAGTAGTGAGTCATGTGTGCAGACTATCTCGTAAACGTTTACTCACGTCCCATGCCTCTGCAGCGCGTA
**13**	365	1:526924-527218	ATCACGCTATCGCTATCACTACGATTCTCGAGTGGTTCGACTCGGAGCGCATACCCTCCACCCGCAATGGGACCCCATCGCATCCCGGTGGGTTGTCGGAGATTGCGCGTCAGAGGCTCGAAGAGCGCAGGAGAGCGCGAGAAGGTATCACCGCCAAGAACCAACCTCGTGAAGACGGACCGCGCGGCCTCGGTGACTTCCAACGACGACTGAATCGGGATCAGCCCTACGACAGGCGAAGAGACGGAGATCGCGGCCGGCGCGAATGGGACGCAACACCACGCTCGGAGCGCAGTCGCCGCGGAGAGGATGCGCCGAGTGTGCGCATACCGAACGCCTCTTGGGACGCCACGCCAAGACGACCC
**15**	1		G
**17**	65	14:109789-109725	CATATCGTCAACATGACCGTGATCGTAATGGATCCTTCTCGGGCGCGGAGGCGACGCAATGTCAT

**18**	396	1:2357208-4663166, 4:1677796-1780190, 22:178337-178283 18:45380-45346, 24:228890-228970	GGACCGATGTTGTACTGGTGACTCGTGTACTACCAAAGATTTTGCTACCCTCCCTCCTCCGACACCAGCTCCACGCTCCAAACTCAAGTTAGCGCAAACGCACACAGAAACTAGCGCGAGTGCCGTAACACTCCCAAACTAACACCGACGTTCTCGTCACCTAACCCTCGCCCGCCGTGGATGTGTTTGAGTTGCCCCAGCGTAACGCTGTGATAACCGACAACATTTCTCGATCCCAAATCTCGTACCCTTTTTGTGCGGGCGTTAAGGAATGTGATAGGAAGGCAATAGGTTTCCATAGTCCGTTCTGACACTGCGAGAGCTCAGCTCCAATCGCAAAGTTCGAGGCATCGCATTCAACTTTAAAGTGCGGTCGACTACCTCGAGGAGGGCCTG
**20**	91	13:889321-889231	TCGGAGTCCTATGGCCGTGGATACTAACACACAAGTTAATTGGCATATAACCCGGCCTTTCTAACCTGCCCTAAATCAGATACCTTTGATG
**21**	280	29:103501-103778	TCCGACGCGCCTGCGCACCACCTCTTCGTCCTCCTCCCGCCCCTCGCCGCAGACGAGACGCACCTCCCCGAGCCCCTCGTCGTCCTCCAAGTCGCGCTCGAGGGCAGCATCTCGCGCCAAGCGATCATGGACGGCCTGAGCCGCGGCTTCCGCGCGGGCGGCGATATGATCCCCTGGCTCGTCGCGCAGCAGTTCCAGGAGAGCAAGTTTGCGATGCTGTCGGGTGCACGCGTGGTGCGGATCGCGACACATCCGGACTACGCGAACATGGGATACGGGG
**22**	58	41:122909-131308	TTCTCTCCGATGCAGACAACCTTCGTAGCGATATCGCTCTACCCTAAGGTTCAAAGCC
**24**	177	mtDNA	TCTTCAGTTTACACTTAGCTGGGGTATCTTCTTTCTAAATATACTTTATATTATGGTTTAAATTCAGATTTTATGTTCTCCATCATTATTTAATCTATGATAACTTTTGACCGTAAATCGGGGAGAAACCACATTCATAGAATATAATCCTAAATTAGTTAATGTAATATTTAAATA
**25**	115	mtDNA	TCGTTAGTGGAATTAACCTAATGTTTAACAACATCGCGCGAAGGGTGGCCTAAGCGGCGGCTACTTCCGCTTGGATGGTACGATGACGATCAACAAGCGGCAGAAGTTGGTCGACC
**26**	71	17:125095-193621	TCGACCCCTGCACTCTCCAACGTCGCCACCAACGTCGCCTGCACGTTCTGTGGTCGCCCCAACCACTCAGA
**27**	351	31:92358-92364, 9:1486283-1486321	GACCTAGCGCTGCGAGCGGGCGTGGCATAAGTAGTTGGCCACCTGCCCGAAATGGTAAGTATAAGCGCTCGTATGTCAAGCATGTACAGCATTGGCCTCCAGACAAACGCGGTGACCATAATTTTGGCATCGGTGTGCATGCTGAATGTATATACACCTATCCACATTTTGAAAAGGCTCCTCAAACTCCCCAAGACGTGGTACATAGAACCAGCTGCAGCCAAACGCCTAGGCGCTAACTGAGAATCGGCGCTTAGTGCATGCAATCCTGCCTATCCCGCATGAGTCCCGCATGCCGATGCGCCCTGCGATACATGAGCAAAGCGTAGTCTGGTTGGCACGCACCTCAGG
**30**	743	14:173743-173691, 7:698848-698809 10:317802-1406807, 15:351658-351762, 1:3494927-3495000, *E. coli* DNA, 13:4955-5023	CGGCTTCCGTAACAGCCTTCACATGGACAAGCTCGAGCGAATCGCAGCTCCGAGTCCGAATCGTCGATGCCCTTATGAGCCGAGGGTTCATCCTTGAGTACGCGAAGAAGGCCGCGTTCTACGTGTTCCACGTCGCGCCCGAGTTCCTCTCCGCCGCGGTGCTCATGGCGCTCAACGCGCGCCGGGGTGCATGGAGTTAATGGACTCCGAGAGCTCGGAGCTGCTCGACTTGTCGATGCAGCATGAGTCGATCCAGAGGTAGCGATAGCCGTTCTCTCGCGCGACACGGCAAGCTTCGCGGATCTTGGGAGAAAGCTTCGGATCGTGCCTGAACCAGCATTGGTAATTTCGACATCGTCAGCAGGAAGTGGTGCACTCACTTCGTCGCTGCTTCCATAAACGACTGCAAGAAACGTGTTAGCTTGCTGCAGGCAATCGGCAAAGAAATACACTTACGGCGCGAAGAAGAGGCGCAGGAAGACGCCGCCAATGCCCAATATGGGCCGTAATAGGGCGCAGATACGGGTCTACTTTCTGGCTTAAATCGCCAGGCAGGAAGCCCAGTTTCTCACCGGCTTCGACCACTGCGCAGTTTTCAAAGCATCATTCAGTTTACATCGGACGTACACAATTTCTAGGCAACGGATAACAACTCACGCCCATGTTGCTGTAGACAATGTCCTTGTGGTCCTGCTATTTATGCTCACCCGATGGGTTTTCAGCCGAGAAAGTGCGTCCGAGGA
**4**	226	17:916595-916710, 16:754081-754183 56:27619-27719, 20:303312-303352 7:57089-57050	AGGGCGAGTCGTTGAAGGATAATGCTATGCAGGGCCGATCCCCTGTTGGTGGCAAGCGGGTCATCGGGAAGGCAGTCGCCAAGGGAGGCATGTTGCTCAAGGGTCGGAATCCACAGGCTGTGAGCTGAAACTCCCTGCAAGATGCAAGAAGCGGACGCGGAGTCACAGAACGCGGCAGAGAACCAGGACCAGGACCAGAAACCACACGTGCGCCGGACTCGGCCGCCCGATCTCCATGCGGAAGCGTACGCCCTTCGCGGCGGCGAGGAAGCCGTCCTCGTCGCGCTCCCGCCAGTCCGCGAGCGCGCAATGTCCAGTCGAGGATGACAGCAACACCCCATGTACAAGTGGGATGACTGGCAGTCCCTGGACCGAGCCATAGAT
	384	40:172409-204900, 36:227614-227502	GATGGCCTTCCTCTCATCTCTGCCCGAGAATAAACCCATCCGCACGGCCTCGAGCAATCCCGCCCGCTCGACGTCCAGGCTGAAGCCGAAGCACTGTTGATGCGTGGCCGTCTCTCAGCGGCCAACCTTCGTTCCTCTTTGCCTACCACACGTAACCTCTCCACATCGCAGCAACGATATCGATACATATCGCCCTTATACTTTCACTCCACACTTACAAGCACAT
**19**	84	18:714262-714179	GGGCCCGTCTCTCCGCGGGCCCCTCAAGAGTCGGAGGAACCGCACGCTGCTACCTTCGCGACTGTCGTAAAGCGCATGGATGCA
	449	mtDNA	CGCCGCCGGCTAACGGCCGTCGGCGACCAGACCGGTCCACACCTCCTCTATACCTGTTATTTTCGCTAAGATGTTGAACTATTCCTCAAATAAGATTGAATTGTACATATATAAATTAAATATGGCTTCTTTTAAAAATAATTTTTTAACCAAATAAAAAGGTTAACTAAAGAACTTTCACAATTATGTGAGATTTTAAAAAACAAATTCAATCTTACAGTAACAATACACTCAGGAGGAAAAAATAAAGGATATACTTTATTAAAACAGCAAAATTATCTTGCCCATATTTTAACAATGCTTGTATAATAGGCATATTTTTATTTCTTTTGTTTTTTAAAAAAGTAGTGTTTAAATAATTTCTTACTTTTTTTAAAAGAATAACAAAAAAAAAAGATTAAATATTATGTTTATCAATTCATTAAGTGCAAGAAACTATGAAATGGTAG

*location of the insert DNA sequence in the genomic DNA of *Ganoderma lucidum* 260125-1 deposited in Mycocosm (mycocosm.jgi.doe.gov/Ganluc1/Ganluc1.home.html).
