# Supplementary material for: Simultaneous gene editing of both nuclei in a dikaryotic strain of Ganoderma lucidum using Cas9-gRNA ribonucleoprotein
Source: J Microbiol. 2025 Jan 24;63(1):e.2409006. doi: 10.71150/jm.2409006 (PMC13577092; doi:10.71150/jm.2409006)
Supplement: Supplementary file 2 [file jm-2409006-Supplementary-data-S1.pdf]

Supplementary Data S1. *MIP1* sequences of *Ganoderma* species.

>OZ026792.1 Ganoderma resinaceum genome assembly, chromosome: 1

AATGGCACGATCGAATAGGTAGGCATAGTAGGTAGCTCCATAGCCGAAAAGGTGTCCAAATTGGGTCTGCCACGACGTTCCAGGAACATATGGAATCAACCCGTGGGCTCCGTAGAGATCTGCTAAAGCTGAAGTAGAACTGAAACCCGGTGTTGTGACGATGGGCGAATG
ATATATTTGATCCAACATAGCAAGGAGAATCTGAGTATGAGTATCGATGGAACGGCAGGAGTCGTGTTGATGGTCGCTTGTTGGGCGGATAGCAAATGAATTGTCATGGCTGAAGAGCGAGAAGACCGCGGGGGAGCTGAGGAAATGCTCCATGAGAATTGAAGGAAGCTC
GACAAAATCTGTCGCGCAACGTGTGCCGGAAACATTCTGATACTCTGTCCGCCCTATCATTGCTTTGTAATATGCTATTAGATCTCTGTCCTCGCAGTTGATGTGACCACTTACAATGCATAGCGTGACCCATCTCGTGAAACAGTGTCAGCACTTCGTGCCACTCGAGAG
CGGTTGGACCGTGTCCCACAGTTGGCCGTACGAACTCGCAGAGGAGAACCACCACAGGGATGTGAAAAGTCGCGTCCCCATCAACAAGTCGATGGCACGCCCCGGCTTCGAACTCCTCTGATAATGCGACGATGGCGTGGTCGTGATCATTGGCGTGGTTGAAATCCCCCA
GCGTGTCGTCGTTGTCCGTCCTACGAGAACACCGCACGGTATAGTGTGCTGCGCCGCCCGGTTTCCCTACGCGTACAAAGAGATCGGCGTAAATCCAGCCGAGAACACCTAGCTCCTCGTCGACGACCTCAAGTTTCCGGATGTCGGGGTGCCAAACCTCCCCTGGTGAGA
CATCGCAGAGTCGCAGTGAAACGCCAAAAATAGCCCGGAACAGTCGCGAAAGTGCCATGAAGACGGTGCCAAGGGTGAGAACGGGAAGGGCGACAGGGGGGGCGGGAGGCTCTGGCGGACAGTAGTAGTCGCGATCCCAGGCTTGGACGATTGGGAATGAAGGAGTGTTTA
GATGCGCCTGTTTGCGCATGCTGAGGTTACGCAGAGCTTGTTTGGCATAAGGCTGGGTGTGGTTCATCAAAGCATCCAGGAAATGTTGGATGTTCTCTGATAAAGCGATCAGATCCATGCGACTAAGAGAGAGAAACTGGTGGAACTACCTGGACTCTTCGCCATCTTGTC
TGTAAGCACCATATGAGCGAAACTCTCCTTCCCAACTAATTGTGCAATCTCTCCACGGGCGCGCAAGAGGTGCTCGAGAACTTCTACCTGCTCAGGGGAACTATAGTTCGCTGCCATATAAACCTTCCTCCGAGGCTCTTCGGCGGGAGCAGAGCGCATAATCATTTGAGC
CTGAAGCGAACCGGGGTAAACCAGGAGATCTCGTTTGGTGACCCGAGCCTGGAGACGTAGCCTGGCGCCCATGCCTAGATCTTTGACACCCTCCAATTCTGACCACTGAATTCGTGCAGGAGGTCGTGGCGCAGCAGTCTCATTCAGGAACTGCCGACCTGACACGATGAT
ATCGGTGGATAGTGAAACGAAACGTTCACGCTGGGGAGGAGGGAGGTCTATGCCAGACTTCTCGAAGTCGCGCCAGAAGATTAGTGCTGTCTGGTACGCCTCGCGACTGAGGGACTTCACAATTTCCTCGTCTGACAAGACATCCCGTAAGACCTGCAGGAAGACGCAAAT
AAGATACTTCAGTTTAAGCCAAATTTTATAATATAGGAGCAATGCGCACTTCATACAAGCCGACGTTCGTGTTGAGGATATTCATGAACTCGCATAGCTTCTCATAGACCTCCTCGGCACTCTGGATCCACGCCTGGTCCGGATGGGCGTTCCTCAACAGCTCCGCAAGGT
CGATGACGCCGCACAGCATGTCGCTCAGCCGGTCCAAGTTCTTCACGACCTTGAACAGCTCGTCCCGCGACTCCCTCGCCCTGAGAATCCGGTCCGTCAGCAGTTGCGCGCGGCGAATGGTAGAGTCGGCGAGGGCAACGAAGGCGGCCGGAGTGGTGAGCATGTGGTGTC
CGAAGAGGCCGGTTGGCGAGGTGAGCTTCTGCGGCGCATAAGGCTGGTCGAAGAAAGCTATGAGGTCCGTGTCATAGACGGAAGCTAGCTGTGTAGCAGAGGTGGTGACCGCTCGTGTAATGGCCGCATCCCTGGCCAGTCGCCCTGTCCTGTGACAACCACGGAAGCGGT
ACGATGGTTTGGAGGCCGTGGAGAGAACATTCTTGGTCACCCGGGCGAGCATTC

>Gansi1 scf_2:2492072-2514338 5'pad=0 3'pad=0 strand=+

AATGGCGCGATCAAATAAGTACGAATAATATGTGGCCCCATATCCAAAAAGATGGCCAAACTGCGTTTGCCATGACGTGCCGGAGACATGGGGAATGAGGCTCTGCTGTGCGTACAACTGTCCTAAGACGGCGGTGGAGTTAAAGTCCGGCGATAGGACCAATGAAGAGTG
ATATGTTTGGTCGAGCATCGCAATGAGAATTTGGGTATGAGTATCGATAGAGCGGCAAGGGTCGTCGAGAGGAGTGCTTGTGGGCCGACTGGCCAACATTCTGTTGCGGTCGAAAAGCGACAGGACGATAGGAGCATTGAGGAAATGTTCCATGAGAATAGAAGGCAGTTC
GACGAAATCAGTTGCACACCGTGTGCCGGAAACATTCTGGTATTCTGTTCGGCCAATCATCGCTGCAAAAGATAATGGTGAGCACCCGATCATATGTACAGCCAGGTCCACTTACAATGCATTGCATGACCCATCTCATGGAAAAGAGTGAGCACTTCGTGCCATTCGAGA
ACCACAGCTCCTCGTCCAACTGTCGGTCGCATGAATTCGCATAGAAGGACGACAACCGGGAGTTGATACGTCACATTCTGGCCAGGGAGCTGAATGTGTTTCTCTGCGTCAAACTGACAAGAGGGTTGGACCAGTAAAGGATCCTGGTGATTTGCATACTTCAAGTCGCTC
CTCTCGTCGTCATCGTCGGTCCGTCGCGAGCAACGCACCGTGTAGTGCGCCGCGCCACTTGGTTTGTCTGGTCGCGCGAAGACGTCGACGTAGATCCAACCAAGCAGGCCGAGGTGGTCGTCCACAACCTCCAACTTCCGCACATCAGTGTGCCACACCTCGCCGGTCGAA
ACTTCGGAAACACGGAGCGACACGCCATAAAGAGCCCGAAACAATCGAGATAGCCCCATAAGCACCGTGCCCAGTGTGAGGGGAGGAAGAGAAATGGGAGGAGTGGGAGCCTCCGGCGGACAGTAGTAGTCACGGTCCCAAGGTTGAATGAGAGGGAACGGAGGTGTGTTC
ATATGAGTTTGTTTTCGTATGCTGAGCGTACGCAACGCACGACTAGCGCGCGGTCGTGTGTGGTTCATGAGGGAGTTTAGAAAATGCTGCACGTTCTCTGTCCAATCTGATCAGCGTCACCGCAAACACTGAACAAGCAAATAATTACCTGGACACTTCGCCATCTTGTCT
GTGAGCGTCATGTGAGAGTAGCTTGCCTTCCCCACCAGCTGCGCGAGCTCGGCACGAGCTCGCAAGAGCCGCTCGAGGAGGTCTATCTGCTCCGGTGTGCTGGAATTTGCAGCCATGTACACCTTTCTCCGTGGCTCTTCAGCCGGTGCAGAGCGCATGATCATCTGTGCT
TGAAGAGAGCCAGGATATACGAGGAGGTCTCGTCTGGTGACTTGAGCTTGAAGGCGTAACCTTGCACCCATGCCCAGATCTTTGACGCCTTGTAGCTCCGACCACTGGATTCGAGCGGGTGGTCGTGGCGCGGAAGTTTCGTTGAGAAATTGCCGACCGAGGACCAGGATC
TCTGTTGACAGTGAAACGAAACGTTCACGCTGCCCTGGTGGAAGGTCTATGCCAGACTTCTCGAAGTCGCGCCAGAAGATGAGTGCTGTCTGATACGCCTCAGAGCTGAGGGACTTCACGACCTCGTCGTCTGCCAAGACGGCCCGCAACACCTGGACGAACACATGAACA
ACGCTTCAATTCACAATAGAATTTGAAAACAAGTGGCAATGCGCACCTCATACAAGCCAACATTTGTATTGAGGACATTCATAAACTCACACAGCTTCTCGTACACCTCGTCGGCACTCTGGATCCACGCCTGGTCCGGATGTGCATTCCTAACCAGCTCCGCGAGATCTA
TGACGCCACACAACATGTCGCTCAGCCTGTCCAAATTCTTCACCACCTTGAACAGCTCGTCCCGCGACTCCCGCGCCCGTAGAATCCGGTCCGTCAGCAGTTGCGCGCGGCGAAGGGTAGAGTCGGCGAGGGCGACGAACGCGGATGGGGTGGTGAGCATGTGGTGTCCGA
AGAGTCCAGTCGGCGAGGTTAGTTTATGGGGGGCATAGGGCTGGTCGAAGAAGGCTATGAGGTCCGTGTCATCGACAGAAGCGGGCTGGATGGCGGAGGTGGTGGCTGGTCGCGAAAGGTTCGCATCCCTGCCCAGTCGCCCTGCCGTGAGACAACCGCGGAAACGGAACA
GTGATTTGGCGGCCGTGGGGATAGCATTCTTGACGACACGGGTGAGCATTC

>Gansp1 scaffold_1:2497062-2519579 5'pad=0 3'pad=0 strand=+

GATAGCACGGTCAAATAGGTAGGAGTAATATGTGGCTCCGTAACCGAAGAGGTGGCCGAATTGAGTTTGCCATGACGTTTCTGGGACGTATGGTATGAGTCCACGAGTCCGGTGGAGGTCGGCCAAGGCGTCAGTGGAGTTGAAAGTCGATTCGAGGACAGAAGGAGAGTG
GTATATTTGATCAAGCATGGCAAGAAGGATCTGAGTATGCGTGTCGACGGACTTGCACGGATCTTCCTGGCGACTTCCCGTCTCTCGGATACTGAACGTCCCATCACGATCGAAGAGAGAAAGTACCGTAGGAGAGTTGAGAAAATGCTCCATGAGGATAGAAGGGAGTTC

GACGAAGTCAGTCGCACACCTCGTCCCCGAAACGTTCTGATATTCCGTTCGACCGATCATAGCTGTTATTGGGTTTTGTCCATCAATAACATTATTTTTCAAGGAGGAAATAACTCAATAACTCACAGTGCATTGCATGACCCATTTCGTGGAAGAGCGTTTGGACTTGGT
GCCATTCCAGAATAGTAGGCCCATGCCCGATGGTCGGACGCATGAATTCACATAGGAGCACCACTACAGGAAACTGGTATGTGGCGTTCTGGTTGGGAACATGGAAATGCTTTCCAGCTTCGAAGTCGACTGAGGGTTGTATAGCCGAGCGATCATGCTCGTCGGCGTGCT
TCAAGTCGCCCGATCCGTCATCGTCGTCTGTCCGTCGCGAACAACGAACAGTGTAATGTGCAGCCCCACCAGGTTTCCCGGTGCGCGCGAATACGTCGGCGTAGATCCAACCGAGTAACCCCAACTGGTCATCAACCACCTCCAGTTTCCGAACGTCTGCATGCCAAACCT
CGCCAGAGGAGACCTCCGACAGCCGTAATGAAACACCATACAGTCTCTGCAATAATCGAGACAGTGCCATGAATACGTTGCCCAAGGTAAGAGACGGTAGTGACATTGGGGGTGCGGGAGGCTCCGGTGGGCAATAGTAATCACGATCCCAGGCTTCGATGGTGGGAAACG
GCGCGCTGTCCAAGTGTGCATGCTTACGCATGCTGAGCACTCGCAAAGCTCGTCTAGCGTGGGGCTGCGTATGGTTCATGAGCGTACTGAGAAAGTGTTGAACGTTATCTAGAGCAAAAGGGCCCACGATCAGTAAAAGACATTCGTGAGTGATATAGACGGAAGTACCTG
GACTCTTTGCCATTTTGTCCGTGAGCGTCATATGTGCAAAGCTCTCTTTCCCTACGAGACGCGCGAGCTCTCCCCGAGCGCTCAAGAGTCGCTCCAGAAGCTCTATCTGTTCAGGGGTGCTCGAGTTCGCTGCCATGTACACCTTTCTCCGTGGCTCCTCCGCGGGGGCAG
AACGCATGATCATCTGAGCTTGAAGAGAACCAGGATACACGAGGAGGTCTCGCTTGGTGACCTGAGCTTGGAGACGTAGCCTTGCGCCCATGCCCAAATCCCGCACGCCTTGCAGCTCCGACCATTTTATCCTAGCAGGGGGCCGTGGGGCCGCGGTCTCGTTGAGGAATT
GCCGACCGAGCACAAGTATTTCTGTAGACAACGAAACGAAACGTTCGCGCTGCCTTGGAGGGAGATCTATACCAGACCTCTCGAAGTCGCGCCAGAAGATGAGGGCGGTCTGGTATGCCTCGGGACTCAGGGACTCAACAACTTCTTGGTCTGACAAGACGTCCCGTAACA
CCTGCACATAGATGCATGCAGAAAACATTAGCCACAACATGAGGAATGTAAAACGGCCATGCGCGCGCACTTCATATAAGCCGACATTGGTGTTAAGGACATTCATGAACTCGCACAACTTCTCGTAAACCTCTTCAGCACTCTGGATCCATACCCGGTCCGGATGCGCGT
TTCTCAACAGCTCTGCGAGGTCGATGACGCTGCATAGCATGTCGCTCAGCCTATCCAAGTTCTTCACGACCTTGAACAGCTCGTCCCGCGACTCCCGCCCCCGCAGAATCCGGTCTGTCAGCAGTTGCGCGCGGCGAAGGGTAGAGTCGGCGAGGGCAATGAAGGCGGCTG
GAGTGGTGAGCATATGGTGTCCGAAGAGGCCTGTCGGCGAAGTGAGCTTCTGGGGAGCATAGGGCTGATCGAAGAAAGCTATGAGGTCCGTGTCATCGACGGAAGCTGGCTGTATAGCAGAGGTGGTGACCGCTCGCGTAATGGCCACATCCCTGGCCAGTCGCCCCGTCC
TATGACAACCGCGGAAGCGGTACGGTGGTTTGGAGGCCGTGGAGAGAACATTCTTGGTCACCCGGGCGAGCATTC

>Ganluc1 scaffold_1:2657111-2651231 5'pad=10000 3'pad=10000 strand=+
GATGGCACGATCAAAGAGGTAGGCATAGTAGGTAGCTCCATAGCCAAAAAGGTGTCCGAATTGGGTCTGCCAGGACGTTCCAGAAACATATGGAATCAACCCACGGGCTCCATAAAGATCTGCCAAAGCCGAGGTAGAACTGAAACTCGGTGTAGCGGCGATGGGCGAATG
ATATGTTTGATCTAACATAGCAAGGAGAATCTGAGTGTGGGTATCGATGGAGCGGCAGGGGTCTTGCTGATGATCGTTTGCTGGGCGGATAGCGAATGAATCGTTATGTCTGAAGAGTGAGAGGACTGTGGGAGAGCTGAGGAAATGCTCCATCAGAATTGAAGGAAGCTC
GACAAAATCTGTCGCGCAACGTGTGCCGGAGACATTCTGGTACTCTGTTCGGCCAATCATAGCTATGAAGGGTTGTCAATCTGTCGAGAAGACACAGCGACCAAAGGACCTAGATGGACTCACAATGCATGGCGTGGCCCATCTCGTGGAAGAGTGTCAACACCTCGTGCC
ACTCCAGGACTGTAGCTCCACGCCCGAGAGTCGGTCGCGCGAATTCGCATAAGAGGACTACGACGGGTAATTGGTATGTAACTTCGTGACCTGGGAGTGAGAAACGGCGCCCCGTTTCGAATTTTTGGGAGAGCTGTGCTCTTGTTCGAGTGGGTTCGTCGATGCCTTCCA
AATCACGttgttcgtcgtcatcgtcggtcCTGCGCGAGCATCGTACGGTGTAATGTGCAGCACCGCCAGGCTTGCCCAAGCGCGTGAATAGGTCAGCGTATATCCAGCCCAACAAGCCCCGCTCCTCGTCAACGACCTCCAGCTTGCGCACATCCGTATGCCAGACCTCGC
CGGGGGACGCATCGGAGACCCGTAGCGACACACCATAAAGAGATTGGAACAATCGTGAGAGGGCCATAAAGACCGTTCCGAGGGTGAGTGGTGGGAGGGAGACAGGGGGGGAAGGCGGCTCGGGAGGACAGTAGTAGTCGCGATCCCATGCGTGAATGACGGGGAAAGGAG
GAGTGTCAAGGTGCGCCTGTTTACGCAAGCTGAGTGCTCGTAGTGCTCTTCTGGCATAAGGTTGTGTGTGATCCATAAGAGCATTCAAGAAATACTGGACATTTTCTAGCCATCTGAATCAGTACGCTGCGCTGAAGTATACCTTATACGAAAGGCACACCTGGACTCTTC
GCCATTTTGTCGACTAATGTCATACGCGCGAAGCTTTCTTTCCCAACCAAATGGGCGAGCTCTCCCCGTGAGCGCAAGAGAcgttcgaggagctcgacctgCTCAGGTGTGCTTGAGTTCGCGGCCATATAGACCTTTCTCCTGGGCTCCTCCGCGGGTGCAGAGCGCATG
ATCATTTGAGCCTGAAGAGATCCAGGGTACACAAGGAGGTCTCTCTTCGTGAACTGCGCTTGGAGACGTAGTCTTGCGCCCATTCCCAGATCCTTAACTCCCTGAAGTTCCGACCACTGTATCCGGgcgggcggacgaggcgcaGCAGTCTCGTTCAAGAATTGTCGACCG
AGCACGAGAATCTCGGTCGACAAGGATACGAAGCGTGCACGTTGTGCGGGAGGTAGGTTTATACCGGACTTCTCGAAGTCTCGCCAGAAAATGAGCGCCGTTTGGTACGCCTCAGGACTGAGAGACTGCACAACTTTCTCGTCGGATAGGACGTCCTGTAACACCTATATA
CCCAGTCGACTTTAGTTTGAGGGAGCGGGATAAATGCAAATGATGCGCACATCATACAGGCCGGCATTTGTGTTGAGGACATTCATGAACTCGCACAGCTTCTCATACACCACCTCGGCACTCTGGATCCACGCCTGGTCCGGATGCGCGTTCCTCAAcagctccgcgagg
tcgatgacgccGCACAGCATGTCGCTCAGCCTGTCCAAGTTCTTCACGACCTTGAACAGTTCGTCCCGCGACTCCCGGGCCCGCAGAATCCGGTCCGTCAGCAGTTGCGCGCGGCAAAGTGTCGAatcggcgagggcgacgaaggcGGATGGTGTAGTGAGCATGTGGTGA
CCGAAAAGTCCGGTTGGCGAGGCGAGTTGCTGCAGGGCATAGGGCTGATCGAAGAAGGCTATGAGGTCCGTGTCATCGACGGAAGCTGGCTggatggcggaggtggtggccgCCCGCGTAATGGTCACGTCCTTGCCCAGTCGGCCCGTCCTGAGCCAACCGCGGAAGCGA
AACGGTGGTTTGGAGGCCGTTGAGGCCGTGGAGAGCACATTCTTGGCCACCCGGGCGAGCATTC

Supplementary Data S2. Sequences of PCR amplicons

>M1 (1,231-bp amplicon)
GAATGCTCGCCCGGGTGCGTTTCCGCGCCACTTGTACTTGCGCCCTGAGTCGTCCTTGAGCGACACATCGCTAGCAGCGCGCGCATGAGGTCAGTGAGATTGTTTCGACTCTGCCCGTATGAGAGCGCGGAGCTCACTCCTTCAGGTAGAACGGTACGAGGCTCGTGTGTA
GCCAGTCGCGCAGGGACGTGGGCGCCTTGTTCCCGAGTGTGACCTTGTCAGGGAGGACATCCCGCCACTCAAGCGACGCGAGTTCCTCCTCGTCTGCGTTTAGGACGTGGGTGACCGTGGCATTGCCATGCGTGGTGTGCACGGTATAGAGCACCTTGTTGTCTTGGTCAA
GGATGGTGGTGTTCGAAGGGTTGTCGCGGGAGAGCGCGAGCGTGACTGCGATTGGGGCGGACATGGTGTGTCGGCTGGGGAGTTGGCTGAGAGAAATGGTGAGAGGTGCTTCTGCTGAGACCGGTCCAGCGAGGCTTTATGTACTTCCCCAAGGAGGCACGGCTTGACAGA
GCACTGCACAGGCTGCCTCAGCCTGTCATTTCATGCGACCTGGAGGCGCTTGCGCGCGCCACAGGCGCATGTGCAAGCAAAAGCTGCTGATCATGGGTCCCATAAGTGTATGCATGCAGGAAGGACCTGGAAGGACTCGGGGATCTGCTTGGACTCGACGATACTCACTAC
TTACGTTCTACGCCTGCGTCCTCGAAACAGGGCCCATCAGGATTGCCTTTGGTCAAGTGCCGGTGCTTGGACAAGCTGTGAGAGACCGAGGTCAACACTACGGAGAATCGCCCCGAGTTCAAAGACCGCACCTGGAGTCGAACGCACGGATGACGGGCTGCAGATCGTGTT
GAACTGGCGCCCAGCCACCGGACACGGGCTTCGGTCCGTGGTTCCGGAAAACCTTACTCGCCATTGTAGAGTAAGGTTTTCCGGATGGTCGGTCCGAACACCTGGCCTTTAGCCGCCCTGTAGCCCAATCGGGCAACTATACCGTACGGTCACCGGCTACCATAAGTAACT
GGTACTGTGATCACAACAGTGATCACACGGACTCTTATATAATCCTGTACTCTACAGTAGTATATAAGACAGCTCTGTAGGAGCTGAATTGACAACTCTTTTAACTCCTCCCATCACGTACGCTATTGTGTACCGCAAGCCTCTAACACCCACATCCAGGTAAGTATGCGG
AGCGTCCGCTAGAGTTGCACCCGGGCGAGCATTC

>M1 (847-bp amplicon)
GAATGCTCGCCCGGGTGGCGGGGTCCGTGGCACTGGCACCTCGAGCGTCGTGGGGCGGTCCGGCTCCGGGGTGCGCGGTCGTTCCGGTCCGTCCGGGTTCGGGTGCGAGGAGGAAGAGGACGAGGAGGAAGTGGAAGTGGAAGTAGAAGTGGAAGTAGAAGTAGAAGGGGG
GGCCGGGGGAGCAGGGATGGCTGGCGCGTCCTCGCAGAGGCCCTTGATCGCGCCCTCCAGAATCTCGGTGGCGCGCAGCGCGCGTTCGGGCGTCGAGAACTCGTGGTTGTTCATGAGGGAGCTGACAATGCGGCGCACGAGCTTCGGGGCGACCTGTCCTGGCTGTGGCGC
GAGCGGCGACGGTTGCGACGTTCTGTCCTGGTCGAGCGGTGGAGGCGTGGAGACGCAGAGCGTGAAGAATCCAGTCACCGCTCCAGCGACGCATTCCTGCCGGAACGACATGCGTTCCCAGAATTCGTCTGCGGAGACGAGCGACAGCTCGCCCAGAGGACGGGTATCCTT
GTCTTTGTCCTTGTCCTTCTCATCCTCATCGCGCTCCTCCGGTTCTCGCGGCGCCCTCTTCTCCCCGCGCGGCCCAGACGACGTCGGTGCTCGTGGCCGTTTGCGCTGAGGTGAACGTACGCCGCTCTCCGTGGTCTTGATGTACGCAATCTCGTCAATGAACCGCGACTT
CGGGTCGTCGTCGAACCACGGTATGACCATCCCTAGATTGTGCGGCGCGTCCGGGTCGGCGCTCTTCGGGCACGGCAGCGGTACGTCGGTCTGGGAATACGGGTGCCCGTACACCCACTGCACGCTCGCGACGGCACGGCTCGCAGCACCCGGGCGAGCATTC

>M2 *MIP1* (2,272-bp amplicon)
GAATGCTCGCCCGGGTGGCCAAGAATGTTCTCTCCACGGCCTCCAAACCACCGTTTCGCTTCCGCATATGTCTCAGGACAGGCCGACTGAGCAAGGATGTGGCCATTACACGAACAACCACCACCTCCGCCATCCAGCCAGCTTCCGTCGATGACACGGACCTCATAGCCT
TCTTTGATCAGCCCTATGCCCCCCAAAAACTCGCCTCGTCAACCGGACTCTTCGGACACCACATGCTCACCACCCCGTCCGCCTTCGTTGCCCTCGCCGACTCTACCCTTCGCCGCGCGCAACTGCTGACGGACCGGATTCTGCGGGCGCGGGAATCGCGGGGCGAGCTGT
TCAAGGTCGTGAAGAACTTGGACAGGCTGAGCGACATGCTGTGCGGCGTCATCGACCTCGCGGAGCTATTGAGGAATGCGCATCCGGACCAGGCATGGATCCAGAGTGCCGAGGAAGTCTACGAGAAGCTGTGCGAGTTCATGAACGTGCTCAACACGAGTGTTGGGTTGT
ACGAAGTGCGCATAATCATGTATCAGTCAACATTCCTTCACAAATTGAAACGTGCATGCAGGTATTGCGAGATGTCCTGTCGGACGAAGAAGTAGTGAAGACCCTCGCCCCTGAGGCGTACCAAACCGCACGCATCTTCTGGCACGACTTCGAGAAATCGGGCATCGACCT
CCCTCCTACGCAGCGCGGACGTTTCGTTTCGTTGTCTACAGAAATTCTTGTGCTCGGTCGGCAATTTCTCAACGAGACTACGGCCCCACGGCCCCCGGCTCGCATAAAATGGTCTGAGCTGCAAGGCGTGCAGGACTTGGGCATGGGAGCAAGGCTACGTCTCCAAGCTCA
GGTCACCAAACGCGACCTCCTTGTGTATCCTGGTTCCCTCCAAGCTCAGATGATCATGCGTTCTGCCCCCGCGGAGGAGCCACGAAGAAAGGTGTACATGGCCGCGAACTCAAGCACCCCTGAACAGATCGAACTTCTGGAGCGACTCTTGCGCGCCCGGGGGGAGCTCGC
GCGTCTCGTAGGAAAAGACAGCTTCGCGCGTATGACATTAGTCGACAAAATGGCGAAGAGTCCAGGTGTGCCTTTCGTATAAGGTATACTTCAGCGCAGCGTACTGATTCAGATGGCTAGAAAATGTCCAGTATTTCTTGAATGCTCTTATGGATCACACACAACCTTATG
CCAGAAGAGCACTACGAGCACTCAGCTTGCGTAAACAGGCGCACCTTGACACTCCTCCGTTCCCAACCATACAAGCCTGGGACCGTGACTACTACTGCCCACCGGAACCTCCCGCACCCCCGGTCTCACTCCCGCCTCTCACCCTGGGCACCGTGTTCATGGCACTGTCTC
GGTTATTTCAGGGCCTGTATGGTGTATCGTTGCGACTATCGGAGGTCTCCTCTGGCGAGGTTTGGCATACAGACGTTCGGAAACTGGAGGTGGTTGATGATCAGTTGGGGGTACTCGGTTGGATCTACGCCGACGTATTCGCACGCATCGGGAAACCTGGTGGTGCCGCAC
ACTATACTGTCCGTTGTTCGCGACGGACAGATGACGACGACGAATCAGGCGACTTGAAGCACGCTGATGAACACGATCGCTTGGCTATACAACCCTTTGCCGACTTCGAAGCTGGAAGACATTTTCGTGTTCCCAGCCAGGACGCGACATACCAACTTCCCGTAGTGGTGC
TCTTATGTGAATTCATGCGTCCGGCCATCGGGCTTGGGCCTACTATTCTGGAATGGCATGAAGTCTTGACACTCTTCCACGAGATGGGTCATGCAATGCACTGTGAGTTGTCTCCGCCTTGGAAAACAGCGTTGTAACCGATTTAAACCCAATGGCAGCCATGATCGGTCG
AACGGAATATCAGAACGTGTCGGGCACGAGGTGTGCAACTGACTTCGTTGAACTTCCTTCTATCCTCATGGAGCATTTCCTCAACTCTCCTACGGTACTTTCCCTCTTCGATCCCGACGGGATGTTCGGTATTCGAGAGACGGGAAGTCGTCGAGAAGATCCATGCAAGTC
CATCGATACGCACACTCAGATCCTTCTTGCCCTGCTTGATCAAATATACCATTCTCCTTCTGCCCTCGAAACGACTTTCAAATCTACTGACGCCCTGGCCGACCTCTACCGGACTCGTGGACTCGTACCTTACGTCCCGGAAACATCATGGCAGACACAATTCGGTCACCT
CTTCGGTTACGGGGCTACATATTATTCACCCGGGCGAGCATTC
