# Supplementary material for: Simultaneous gene editing of both nuclei in a dikaryotic strain of Ganoderma lucidum using Cas9-gRNA ribonucleoprotein
Source: J Microbiol. 2025 Jan 24;63(1):e.2409006. doi: 10.71150/jm.2409006 (PMC13577092; doi:10.71150/jm.2409006)
Supplement: Supplementary file 3 [file jm-2409006-Supplementary-Table-S2.docx]

**Supplementary Table S2.** Origins of the insert DNAs

| **No.** | **Inserted DNA size (bp)** | **Origin of the insert DNA** | **Inserted DNA Sequence** |
| --- | --- | --- | --- |
| **1** | 502 | *9:1187485-1187628,30:129758-129896  12:826662-826766, 7:1965253-1965345 | TGTTATAGCCGGGTCGTGCTTGTAGCCGGGTCGCATGGGCGGCATAACTAAACTCGGGCGGGTATGCAGCGTTCATCACTAGTACTCTCAGGCGATCTTGCCCGGTTTCTCGTCCGACGTGTTCGCGTGGGCCAATCATGCGCCTCTCTGTCTTATTCCTGTCCCCCTCCCCCTCATCTCGACTTCTCCCTGCTCTCGTCGCTGCCTGACGGGCAGGCCAGCCCATGCAGTTCGCGTACAGAGTAATTCACTCTGGCTCTACGACTGCACCAATGGGTACCATCTGGATATGACTCTCGTGCACGCGCGGGGCGTCCGGGGCGTCGGCTGCGGCGGCGACTGAGGAGGGGAAGGAGAACACGCCGGAGCCGGGGGCGGACGAGGAGAATGAGAAAGAGGAGGCAACTTCGACATTTCCCTAGCGTCGTCTAACGAGTAGTGAGTCATGTGTGCAGACTATCTCGTAAACGTTTACTCACGTCCCATGCCTCTGCAGCGCGTA |
| **5** | 108 | 21:699229-699331 | GCCGATCATTGAGATGCCTGTCCCTTGTCTTGACCACCCACTGCACAGTATTCTTGAAATCCAACATGAGCCTCTGGCCGACAACCATCGATTTCCGCGTTCGGAGCT |
| **6** | 378 | 5:1270291-1520218, mtDNA, 14:646678-646679 | ACCCGGTGTACAAAGGCTACTATGAACTCCCTGACATGATCAAGGCCAAGGGCTCAGCATCTGGCACTCGACTACGTGCTATGGACGAAGAAAATTATTATAAACTTTGATTGTTTTTATTTGTTCTACAATTTTACTTAAAAAACAAGTTGAGTATTCGATATCAAAATATGGAAAAGACTCGTACCAGCGCACCCACTCCTGTGTTGCCACCAAGGAAATGATCGGAAAGCGTTAAATCTAGGGATAGTCACAGGCCTATGTGGATGTCGAATTGACAGGGTTCAGAAATGTTGTCGATTTTGAATTGCGAAAGCCTTGCAACCATCTGGGGACACTGACAAGAGGAAACCATTTAGGGAATACGTTTTTGGTTGG |
| **7** | 540 | 32:54644-54812, 6:1597390-1599980  15:775042-774945, 17:497829-497928  4:1550725-1550633, 8:1500509-1500539 | CAACGAGCTGCTCGCGCGCCTGACGGGCCAGCCGGTGCGCGACCACACGCAGACGAACTCGACGCTGGAGATGCATACAAGCAAGAGGACGAGGCAGCCGAGGTTCGTGAATCCTCGCATGGAGAACACGTGACCGTTTCCCTTCCCCTTCTCCTTCGTAGTATCCCCCCGACTCCCCTTCATCCCACTCGTCCTCCTCTTCTTCGCCCTGAAGGACATCTCCAAGGGCCAAAAGTAAACTTGTCGTGCGGGGAGACCGGATCGTCAGTACTTCGTCCTGACTTGAGTCCCAAATCCAAGCCCCCGTCATCCGATCGTCCGCCTGCCAATCCAGCCCCATCTCGCTCAGAAAGTCAATATGTTGGAATAGGGGTGTTACTCACTGCGAAACTGTTGTCGCACTTTCTCCGGCTGCAGCCAAACGGGCCCAGGGACTCCAAAGGCAGGTGGTACGAAGCCCGTCTCCCGCCCACCTCGCCACTTCGATGAGGAGCTCGCGTTTCTTGTCGACACCTGCCATCGAACGGAGCTCATAGAGCG |
| **8** | 342 | unidentified | CGATCGCTCACCCTTAGAATTAACACGTCGAGATGGCGTCCCCTCGCACCTCCGACTCAGTAAAGAACTCTCGCCCTGTCGACGCGCGAAAGTCCCAATGCCGCACACCCGCCATAAACCCAGCCAAGGCCACGAAGCTAGCAGATCCAGCCGCGAAGGCTGCTGAGCAGGCACAGACCGAGGAGGCGTCGGACTACGGCAGCGACACCTACGTTGAGATAGACAACACTGAGCGGCTTCCTGCGAAGAAGGCCAAGACAATGCCGCCAGCGCGCCGGTCGTCCTACAATGTCATCGCCGGTTGCATTCTGTCCCACGCCGAACCGATTGCTCGGTGCTCGC |
| **10** | 312 | mtDNA | ATTACCTTCTTTAGGTTTAATTAAATTTTGAGAATAGCTTAATTTAGCTTCTAATATTTCATCGCTATTTATTCTATAGAAAAATATTAATTCTTTAATAATAAAATCTCTATTATCAAAATATTAGGATACTCATTTCCCACTAAATTAAATTTAGATAGCTGGGGAATGGATATAATTTCGTATATTTTTTTCTTATTATGTCCTGTCTAAGATCATCTTTAATTATATTAATTTTTTCTATTACTTTTGATAGGAGAGTGTCTGTTTGATGAGTTAAATCTTTGGCCACATGACTTTTATCTTGATAGT |
| **11** | 102 | 8:1428154-1428255 | GGTCAGACCGATCTTGAGCGGGATGCGGAAGCCAGTCGCGCGGCGGACGGGAGTCCAGCCGGCAGGGACGAAGCTGCGAGACTCGTGGAGGACCCGGGGCGA |
| **12** | 502 | 9:1187485-1187628, 12:826766-826662, 30:129758-129896 | TGTTATAGCCGGGTCGTGCTTGTAGCCGGGTCGCATGGGCGGCATAACTAAACTCGGGCGGGTATGCAGCGTTCATCACTAGTACTCTCAGGCGATCTTGCCCGGTTTCTCGTCCGACGTGTTCGCGTGGGCCAATCATGCGCCTCTCTGTCTTATTCCTGTCCCCCTCCCCCTCATCTCGACTTCTCCCTGCTCTCGTCGCTGCCTGACGGGCAGGCCAGCCCATGCAGTTCGCGTACAGAGTAATTCACTCTGGCTCTACGACTGCACCAATGGGTACCATCTGGATATGACTCTCGTGCACGCGCGGGGCGTCCGGGGCGTCGGCTGCGGCGGCGACTGAGGAGGGGAAGGAGAACACGCCGGAGCCGGGGGCGGACGAGGAGAATGAGAAAGAGGAGGCAACTTCGACATTTCCCTAGCGTCGTCTAACGAGTAGTGAGTCATGTGTGCAGACTATCTCGTAAACGTTTACTCACGTCCCATGCCTCTGCAGCGCGTA |
| **13** | 365 | 1:526924-527218 | ATCACGCTATCGCTATCACTACGATTCTCGAGTGGTTCGACTCGGAGCGCATACCCTCCACCCGCAATGGGACCCCATCGCATCCCGGTGGGTTGTCGGAGATTGCGCGTCAGAGGCTCGAAGAGCGCAGGAGAGCGCGAGAAGGTATCACCGCCAAGAACCAACCTCGTGAAGACGGACCGCGCGGCCTCGGTGACTTCCAACGACGACTGAATCGGGATCAGCCCTACGACAGGCGAAGAGACGGAGATCGCGGCCGGCGCGAATGGGACGCAACACCACGCTCGGAGCGCAGTCGCCGCGGAGAGGATGCGCCGAGTGTGCGCATACCGAACGCCTCTTGGGACGCCACGCCAAGACGACCC |
| **15** | 1 |  | G |
| **17** | 65 | 14:109789-109725 | CATATCGTCAACATGACCGTGATCGTAATGGATCCTTCTCGGGCGCGGAGGCGACGCAATGTCAT |
| **18** | 396 | 1:2357208-4663166, 4:1677796-1780190, 22:178337-178283  18:45380-45346, 24:228890-228970 | GGACCGATGTTGTACTGGTGACTCGTGTACTACCAAAGATTTTGCTACCCTCCCTCCTCCGACACCAGCTCCACGCTCCAAACTCAAGTTAGCGCAAACGCACACAGAAACTAGCGCGAGTGCCGTAACACTCCCAAACTAACACCGACGTTCTCGTCACCTAACCCTCGCCCGCCGTGGATGTGTTTGAGTTGCCCCAGCGTAACGCTGTGATAACCGACAACATTTCTCGATCCCAAATCTCGTACCCTTTTTGTGCGGGCGTTAAGGAATGTGATAGGAAGGCAATAGGTTTCCATAGTCCGTTCTGACACTGCGAGAGCTCAGCTCCAATCGCAAAGTTCGAGGCATCGCATTCAACTTTAAAGTGCGGTCGACTACCTCGAGGAGGGCCTG |
| **20** | 91 | 13:889321-889231 | TCGGAGTCCTATGGCCGTGGATACTAACACACAAGTTAATTGGCATATAACCCGGCCTTTCTAACCTGCCCTAAATCAGATACCTTTGATG |
| **21** | 280 | 29:103501-103778 | TCCGACGCGCCTGCGCACCACCTCTTCGTCCTCCTCCCGCCCCTCGCCGCAGACGAGACGCACCTCCCCGAGCCCCTCGTCGTCCTCCAAGTCGCGCTCGAGGGCAGCATCTCGCGCCAAGCGATCATGGACGGCCTGAGCCGCGGCTTCCGCGCGGGCGGCGATATGATCCCCTGGCTCGTCGCGCAGCAGTTCCAGGAGAGCAAGTTTGCGATGCTGTCGGGTGCACGCGTGGTGCGGATCGCGACACATCCGGACTACGCGAACATGGGATACGGGG |
| **22** | 58 | 41:122909-131308 | TTCTCTCCGATGCAGACAACCTTCGTAGCGATATCGCTCTACCCTAAGGTTCAAAGCC |
| **24** | 177 | mtDNA | TCTTCAGTTTACACTTAGCTGGGGTATCTTCTTTCTAAATATACTTTATATTATGGTTTAAATTCAGATTTTATGTTCTCCATCATTATTTAATCTATGATAACTTTTGACCGTAAATCGGGGAGAAACCACATTCATAGAATATAATCCTAAATTAGTTAATGTAATATTTAAATA |
| **25** | 115 | mtDNA | TCGTTAGTGGAATTAACCTAATGTTTAACAACATCGCGCGAAGGGTGGCCTAAGCGGCGGCTACTTCCGCTTGGATGGTACGATGACGATCAACAAGCGGCAGAAGTTGGTCGACC |
| **26** | 71 | 17:125095-193621 | TCGACCCCTGCACTCTCCAACGTCGCCACCAACGTCGCCTGCACGTTCTGTGGTCGCCCCAACCACTCAGA |
| **27** | 351 | 31:92358-92364, 9:1486283-1486321 | GACCTAGCGCTGCGAGCGGGCGTGGCATAAGTAGTTGGCCACCTGCCCGAAATGGTAAGTATAAGCGCTCGTATGTCAAGCATGTACAGCATTGGCCTCCAGACAAACGCGGTGACCATAATTTTGGCATCGGTGTGCATGCTGAATGTATATACACCTATCCACATTTTGAAAAGGCTCCTCAAACTCCCCAAGACGTGGTACATAGAACCAGCTGCAGCCAAACGCCTAGGCGCTAACTGAGAATCGGCGCTTAGTGCATGCAATCCTGCCTATCCCGCATGAGTCCCGCATGCCGATGCGCCCTGCGATACATGAGCAAAGCGTAGTCTGGTTGGCACGCACCTCAGG |
| **30** | 743 | 14:173743-173691, 7:698848-698809  10:317802-1406807, 15:351658-351762, 1:3494927-3495000, *E. coli* DNA, 13:4955-5023 | CGGCTTCCGTAACAGCCTTCACATGGACAAGCTCGAGCGAATCGCAGCTCCGAGTCCGAATCGTCGATGCCCTTATGAGCCGAGGGTTCATCCTTGAGTACGCGAAGAAGGCCGCGTTCTACGTGTTCCACGTCGCGCCCGAGTTCCTCTCCGCCGCGGTGCTCATGGCGCTCAACGCGCGCCGGGGTGCATGGAGTTAATGGACTCCGAGAGCTCGGAGCTGCTCGACTTGTCGATGCAGCATGAGTCGATCCAGAGGTAGCGATAGCCGTTCTCTCGCGCGACACGGCAAGCTTCGCGGATCTTGGGAGAAAGCTTCGGATCGTGCCTGAACCAGCATTGGTAATTTCGACATCGTCAGCAGGAAGTGGTGCACTCACTTCGTCGCTGCTTCCATAAACGACTGCAAGAAACGTGTTAGCTTGCTGCAGGCAATCGGCAAAGAAATACACTTACGGCGCGAAGAAGAGGCGCAGGAAGACGCCGCCAATGCCCAATATGGGCCGTAATAGGGCGCAGATACGGGTCTACTTTCTGGCTTAAATCGCCAGGCAGGAAGCCCAGTTTCTCACCGGCTTCGACCACTGCGCAGTTTTCAAAGCATCATTCAGTTTACATCGGACGTACACAATTTCTAGGCAACGGATAACAACTCACGCCCATGTTGCTGTAGACAATGTCCTTGTGGTCCTGCTATTTATGCTCACCCGATGGGTTTTCAGCCGAGAAAGTGCGTCCGAGGA |
| **4** | 226 | 17:916595-916710, 16:754081-754183  56:27619-27719, 20:303312-303352  7:57089-57050 | AGGGCGAGTCGTTGAAGGATAATGCTATGCAGGGCCGATCCCCTGTTGGTGGCAAGCGGGTCATCGGGAAGGCAGTCGCCAAGGGAGGCATGTTGCTCAAGGGTCGGAATCCACAGGCTGTGAGCTGAAACTCCCTGCAAGATGCAAGAAGCGGACGCGGAGTCACAGAACGCGGCAGAGAACCAGGACCAGGACCAGAAACCACACGTGCGCCGGACTCGGCCGCCCGATCTCCATGCGGAAGCGTACGCCCTTCGCGGCGGCGAGGAAGCCGTCCTCGTCGCGCTCCCGCCAGTCCGCGAGCGCGCAATGTCCAGTCGAGGATGACAGCAACACCCCATGTACAAGTGGGATGACTGGCAGTCCCTGGACCGAGCCATAGAT |
|  | 384 | 40:172409-204900, 36:227614-227502 | GATGGCCTTCCTCTCATCTCTGCCCGAGAATAAACCCATCCGCACGGCCTCGAGCAATCCCGCCCGCTCGACGTCCAGGCTGAAGCCGAAGCACTGTTGATGCGTGGCCGTCTCTCAGCGGCCAACCTTCGTTCCTCTTTGCCTACCACACGTAACCTCTCCACATCGCAGCAACGATATCGATACATATCGCCCTTATACTTTCACTCCACACTTACAAGCACAT |
| **19** | 84 | 18:714262-714179 | GGGCCCGTCTCTCCGCGGGCCCCTCAAGAGTCGGAGGAACCGCACGCTGCTACCTTCGCGACTGTCGTAAAGCGCATGGATGCA |
|  | 449 | mtDNA | CGCCGCCGGCTAACGGCCGTCGGCGACCAGACCGGTCCACACCTCCTCTATACCTGTTATTTTCGCTAAGATGTTGAACTATTCCTCAAATAAGATTGAATTGTACATATATAAATTAAATATGGCTTCTTTTAAAAATAATTTTTTAACCAAATAAAAAGGTTAACTAAAGAACTTTCACAATTATGTGAGATTTTAAAAAACAAATTCAATCTTACAGTAACAATACACTCAGGAGGAAAAAATAAAGGATATACTTTATTAAAACAGCAAAATTATCTTGCCCATATTTTAACAATGCTTGTATAATAGGCATATTTTTATTTCTTTTGTTTTTTAAAAAAGTAGTGTTTAAATAATTTCTTACTTTTTTTAAAAGAATAACAAAAAAAAAAGATTAAATATTATGTTTATCAATTCATTAAGTGCAAGAAACTATGAAATGGTAG |

*location of the insert DNA sequence in the genomic DNA of *Ganoderma lucidum* 260125-1 deposited in Mycocosm (mycocosm.jgi.doe.gov/Ganluc1/Ganluc1.home.html).
