# Supplementary material for: Simultaneous gene editing of both nuclei in a dikaryotic strain of Ganoderma lucidum using Cas9-gRNA ribonucleoprotein
Source: J Microbiol. 2025 Jan 24;63(1):e.2409006. doi: 10.71150/jm.2409006 (PMC13577092; doi:10.71150/jm.2409006)
Supplement: Supplementary file 4 [file jm-2409006-Supplementary-data-S2.docx]

**Supplementary Data S2.** Sequences of PCR amplicons

>M1 (1,231-bp amplicon)

GAATGCTCGCCCGGGTGCGTTTCCGCGCCACTTGTACTTGCGCCCTGAGTCGTCCTTGAGCGACACATCGCTAGCAGCGCGCGCATGAGGTCAGTGAGATTGTTTCGACTCTGCCCGTATGAGAGCGCGGAGCTCACTCCTTCAGGTAGAACGGTACGAGGCTCGTGTGTAGCCAGTCGCGCAGGGACGTGGGCGCCTTGTTCCCGAGTGTGACCTTGTCAGGGAGGACATCCCGCCACTCAAGCGACGCGAGTTCCTCCTCGTCTGCGTTTAGGACGTGGGTGACCGTGGCATTGCCATGCGTGGTGTGCACGGTATAGAGCACCTTGTTGTCTTGGTCAAGGATGGTGGTGTTCGAAGGGTTGTCGCGGGAGAGCGCGAGCGTGACTGCGATTGGGGCGGACATGGTGTGTCGGCTGGGGAGTTGGCTGAGAGAAATGGTGAGAGGTGCTTCTGCTGAGACCGGTCCAGCGAGGCTTTATGTACTTCCCCAAGGAGGCACGGCTTGACAGAGCACTGCACAGGCTGCCTCAGCCTGTCATTTCATGCGACCTGGAGGCGCTTGCGCGCGCCACAGGCGCATGTGCAAGCAAAAGCTGCTGATCATGGGTCCCATAAGTGTATGCATGCAGGAAGGACCTGGAAGGACTCGGGGATCTGCTTGGACTCGACGATACTCACTACTTACGTTCTACGCCTGCGTCCTCGAAACAGGGCCCATCAGGATTGCCTTTGGTCAAGTGCCGGTGCTTGGACAAGCTGTGAGAGACCGAGGTCAACACTACGGAGAATCGCCCCGAGTTCAAAGACCGCACCTGGAGTCGAACGCACGGATGACGGGCTGCAGATCGTGTTGAACTGGCGCCCAGCCACCGGACACGGGCTTCGGTCCGTGGTTCCGGAAAACCTTACTCGCCATTGTAGAGTAAGGTTTTCCGGATGGTCGGTCCGAACACCTGGCCTTTAGCCGCCCTGTAGCCCAATCGGGCAACTATACCGTACGGTCACCGGCTACCATAAGTAACTGGTACTGTGATCACAACAGTGATCACACGGACTCTTATATAATCCTGTACTCTACAGTAGTATATAAGACAGCTCTGTAGGAGCTGAATTGACAACTCTTTTAACTCCTCCCATCACGTACGCTATTGTGTACCGCAAGCCTCTAACACCCACATCCAGGTAAGTATGCGGAGCGTCCGCTAGAGTTGCACCCGGGCGAGCATTC

>M1 (847-bp amplicon)
GAATGCTCGCCCGGGTGGCGGGGTCCGTGGCACTGGCACCTCGAGCGTCGTGGGGCGGTCCGGCTCCGGGGTGCGCGGTCGTTCCGGTCCGTCCGGGTTCGGGTGCGAGGAGGAAGAGGACGAGGAGGAAGTGGAAGTGGAAGTAGAAGTGGAAGTAGAAGTAGAAGGGGGGGCCGGGGGAGCAGGGATGGCTGGCGCGTCCTCGCAGAGGCCCTTGATCGCGCCCTCCAGAATCTCGGTGGCGCGCAGCGCGCGTTCGGGCGTCGAGAACTCGTGGTTGTTCATGAGGGAGCTGACAATGCGGCGCACGAGCTTCGGGGCGACCTGTCCTGGCTGTGGCGCGAGCGGCGACGGTTGCGACGTTCTGTCCTGGTCGAGCGGTGGAGGCGTGGAGACGCAGAGCGTGAAGAATCCAGTCACCGCTCCAGCGACGCATTCCTGCCGGAACGACATGCGTTCCCAGAATTCGTCTGCGGAGACGAGCGACAGCTCGCCCAGAGGACGGGTATCCTTGTCTTTGTCCTTGTCCTTCTCATCCTCATCGCGCTCCTCCGGTTCTCGCGGCGCCCTCTTCTCCCCGCGCGGCCCAGACGACGTCGGTGCTCGTGGCCGTTTGCGCTGAGGTGAACGTACGCCGCTCTCCGTGGTCTTGATGTACGCAATCTCGTCAATGAACCGCGACTTCGGGTCGTCGTCGAACCACGGTATGACCATCCCTAGATTGTGCGGCGCGTCCGGGTCGGCGCTCTTCGGGCACGGCAGCGGTACGTCGGTCTGGGAATACGGGTGCCCGTACACCCACTGCACGCTCGCGACGGCACGGCTCGCAGCACCCGGGCGAGCATTC

>M2 *MIP1* (2,272-bp amplicon)

GAATGCTCGCCCGGGTGGCCAAGAATGTTCTCTCCACGGCCTCCAAACCACCGTTTCGCTTCCGCATATGTCTCAGGACAGGCCGACTGAGCAAGGATGTGGCCATTACACGAACAACCACCACCTCCGCCATCCAGCCAGCTTCCGTCGATGACACGGACCTCATAGCCTTCTTTGATCAGCCCTATGCCCCCCAAAAACTCGCCTCGTCAACCGGACTCTTCGGACACCACATGCTCACCACCCCGTCCGCCTTCGTTGCCCTCGCCGACTCTACCCTTCGCCGCGCGCAACTGCTGACGGACCGGATTCTGCGGGCGCGGGAATCGCGGGGCGAGCTGTTCAAGGTCGTGAAGAACTTGGACAGGCTGAGCGACATGCTGTGCGGCGTCATCGACCTCGCGGAGCTATTGAGGAATGCGCATCCGGACCAGGCATGGATCCAGAGTGCCGAGGAAGTCTACGAGAAGCTGTGCGAGTTCATGAACGTGCTCAACACGAGTGTTGGGTTGTACGAAGTGCGCATAATCATGTATCAGTCAACATTCCTTCACAAATTGAAACGTGCATGCAGGTATTGCGAGATGTCCTGTCGGACGAAGAAGTAGTGAAGACCCTCGCCCCTGAGGCGTACCAAACCGCACGCATCTTCTGGCACGACTTCGAGAAATCGGGCATCGACCTCCCTCCTACGCAGCGCGGACGTTTCGTTTCGTTGTCTACAGAAATTCTTGTGCTCGGTCGGCAATTTCTCAACGAGACTACGGCCCCACGGCCCCCGGCTCGCATAAAATGGTCTGAGCTGCAAGGCGTGCAGGACTTGGGCATGGGAGCAAGGCTACGTCTCCAAGCTCAGGTCACCAAACGCGACCTCCTTGTGTATCCTGGTTCCCTCCAAGCTCAGATGATCATGCGTTCTGCCCCCGCGGAGGAGCCACGAAGAAAGGTGTACATGGCCGCGAACTCAAGCACCCCTGAACAGATCGAACTTCTGGAGCGACTCTTGCGCGCCCGGGGGGAGCTCGCGCGTCTCGTAGGAAAAGACAGCTTCGCGCGTATGACATTAGTCGACAAAATGGCGAAGAGTCCAGGTGTGCCTTTCGTATAAGGTATACTTCAGCGCAGCGTACTGATTCAGATGGCTAGAAAATGTCCAGTATTTCTTGAATGCTCTTATGGATCACACACAACCTTATGCCAGAAGAGCACTACGAGCACTCAGCTTGCGTAAACAGGCGCACCTTGACACTCCTCCGTTCCCAACCATACAAGCCTGGGACCGTGACTACTACTGCCCACCGGAACCTCCCGCACCCCCGGTCTCACTCCCGCCTCTCACCCTGGGCACCGTGTTCATGGCACTGTCTCGGTTATTTCAGGGCCTGTATGGTGTATCGTTGCGACTATCGGAGGTCTCCTCTGGCGAGGTTTGGCATACAGACGTTCGGAAACTGGAGGTGGTTGATGATCAGTTGGGGGTACTCGGTTGGATCTACGCCGACGTATTCGCACGCATCGGGAAACCTGGTGGTGCCGCACACTATACTGTCCGTTGTTCGCGACGGACAGATGACGACGACGAATCAGGCGACTTGAAGCACGCTGATGAACACGATCGCTTGGCTATACAACCCTTTGCCGACTTCGAAGCTGGAAGACATTTTCGTGTTCCCAGCCAGGACGCGACATACCAACTTCCCGTAGTGGTGCTCTTATGTGAATTCATGCGTCCGGCCATCGGGCTTGGGCCTACTATTCTGGAATGGCATGAAGTCTTGACACTCTTCCACGAGATGGGTCATGCAATGCACTGTGAGTTGTCTCCGCCTTGGAAAACAGCGTTGTAACCGATTTAAACCCAATGGCAGCCATGATCGGTCGAACGGAATATCAGAACGTGTCGGGCACGAGGTGTGCAACTGACTTCGTTGAACTTCCTTCTATCCTCATGGAGCATTTCCTCAACTCTCCTACGGTACTTTCCCTCTTCGATCCCGACGGGATGTTCGGTATTCGAGAGACGGGAAGTCGTCGAGAAGATCCATGCAAGTCCATCGATACGCACACTCAGATCCTTCTTGCCCTGCTTGATCAAATATACCATTCTCCTTCTGCCCTCGAAACGACTTTCAAATCTACTGACGCCCTGGCCGACCTCTACCGGACTCGTGGACTCGTACCTTACGTCCCGGAAACATCATGGCAGACACAATTCGGTCACCTCTTCGGTTACGGGGCTACATATTATTCACCCGGGCGAGCATTC
